# Identification of Substrates of Cytoplasmic Peptidyl-Prolyl *Cis/Trans* Isomerases and Their Collective Essentiality in *Escherichia Coli*

**DOI:** 10.3390/ijms21124212

**Published:** 2020-06-13

**Authors:** Gracjana Klein, Pawel Wojtkiewicz, Daria Biernacka, Anna Stupak, Patrycja Gorzelak, Satish Raina

**Affiliations:** Unit of Bacterial Genetics, Gdansk University of Technology, 80-233 Gdansk, Poland; pawwojtk1@student.pg.edu.pl (P.W.); darbiern@student.pg.edu.pl (D.B.); anna.stupak@pg.edu.pl (A.S.); patrycja.gorzelak@gmail.com (P.G.)

**Keywords:** prolyl isomerase, protein folding, heat shock proteins, protein aggregation, RpoE sigma factor, PpiB, PpiC, FkpB, FklB, AhpC

## Abstract

Protein folding often requires molecular chaperones and folding catalysts, such as peptidyl-prolyl *cis/trans* isomerases (PPIs). The *Escherichia coli* cytoplasm contains six well-known PPIs, although a requirement of their PPIase activity, the identity of their substrates and relative enzymatic contribution is unknown. Thus, strains lacking all periplasmic and one of the cytoplasmic PPIs were constructed. Measurement of their PPIase activity revealed that PpiB is the major source of PPIase activity in the cytoplasm. Furthermore, viable Δ6*ppi* strains could be constructed only on minimal medium in the temperature range of 30–37 °C, but not on rich medium. To address the molecular basis of essentiality of PPIs, proteins that aggregate in their absence were identified. Next, wild-type and putative active site variants of FkpB, FklB, PpiB and PpiC were purified and in pull-down experiments substrates specific to each of these PPIs identified, revealing an overlap of some substrates. Substrates of PpiC were validated by immunoprecipitations using extracts from wild-type and PpiC-H81A strains carrying a 3xFLAG-tag appended to the C-terminal end of the *ppiC* gene on the chromosome. Using isothermal titration calorimetry, RpoE, RseA, S2, and AhpC were established as FkpB substrates and PpiC’s PPIase activity was shown to be required for interaction with AhpC.

## 1. Introduction

Cellular protein concentration in the cytoplasm is relatively very high, which leads to macromolecular crowding and exposure of hydrophobic surfaces that can cause protein aggregation [1]. Thus, in vivo, the process of folding can compete with the formation of aberrant aggregates, which can lead to severe cellular defects. Hence, in order to carry out their biological functions, most polypeptide chains must fold rapidly into the stable three-dimensional conformation. This is achieved with the help of conserved group of molecular machines to maintain cellular protein homeostasis. In vivo partially folded polypeptides are bound by chaperones to prevent their aggregation or misfolding and critical slow rate-limiting folding steps are accelerated by folding catalysts, such as peptidyl-prolyl *cis*/*trans* isomerases (PPIs) and thiol-disulfide oxidoreductases (PDIs). PPIs are ubiquitous enzymes, found in all organisms and they catalyze the slow *cis*/*trans* isomerization of prolyl peptide Xaa-Pro bonds. Due to the relatively high energy barrier (14–24 kcal/mol), the uncatalyzed isomerization is a slow process with an exchange time constant on the order of several minutes [2]. Hence, PPIs reduce the energy barrier between *cis* and *trans* states and hence accelerate the isomerization [3]. PPIs were initially identified as targets of two immunosuppressive drugs: cyclosporine A (CsA) or FK506 and were hence called cyclophilins and FK506-binding proteins (FKBPs), respectively, and are collectively known as immunophilins [4]. The third family of PPIs is called parvulins, which are distinct from cyclophilins and FKBPs, since their PPIase activity is neither inhibited by cyclosporine nor FK506 [5].

The model bacterium *Escherichia coli* contains ten PPIs that cover all three families of PPIs. Out of these, six PPIs are present in the cytoplasm. These six *E*. *coli* cytoplasmic PPIs include the cyclophilin PpiB, the FKBPs {trigger factor (Tig), SlyD, FkpB, FklB} and the parvulin PpiC. Although the PPIase activity for each of six cytoplasmic PPIs have been demonstrated, the relative contribution towards the cellular pool has not been reported. Similarly, there is a lack of information about the cellular function of most of PPIs in *E. coli* other than Tig and to some extent for SlyD. Tig is an abundant ribosome-associated protein, consisting of an N-terminal ribosome-binding domain, a PPIase domain and a C-terminal domain [6]. Ribosome profiling studies have revealed that the Tig protein interacts with all nascently synthesized polypeptides with the highest interaction for *β*-barrel outer membrane proteins (OMPs) [7]. Another cytoplasmic PPI SlyD has been shown to be a two-domain protein functioning as a molecular chaperone, a prolyl *cis/trans* isomerase, and a nickel-binding protein [8,9]. However, its PPIase activity may not be required for its function for the nickel insertion in [NiFe]-hydrogenase [10]. Regarding other cytoplasmic PPIs, on the basis of co-purification the alkyl hydroperoxide reductase subunit C (AhpC) has been suggested as a substrate of PpiC [11]. The FkpB also has a two-domain architecture like SlyD. Using pull-down experiments some ribosomal proteins were found to co-elute with FkpB [12]. However, a complete spectrum of substrates with mutational comparisons in PPI-encoding genes remains unknown in most of the cases.

To gain a better understanding of function of cytoplasmic PPIs, suppressor-free Δ6*ppi* strains were constructed and characterized for their defects in protein folding. Several proteins were identified that aggregate in Δ6*ppi* strains, some of which are essential for bacterial growth. Further, in vivo substrates were identified by pull-down and immunoprecipitations experiments using wild-type and putative active site variants of different PPIs. Among various substrates, an interaction of AhpC with FkpB and PpiC was shown and the essential sigma factor RpoE and its cognate anti-sigma factor RseA established as clients of FkpB using isothermal titration calorimetry.

## 2. Results

### 2.1. The Peptidyl-Prolyl Cis/Trans Isomerase Activity Is Required for Optimal Growth

**Figure 1:**
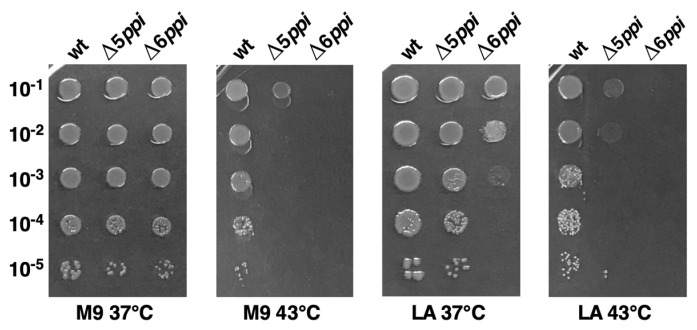
Δ6*ppi* bacteria are unable to grow at either 37 or 43 °C on LA medium. Exponentially grown cultures of the wild type and its isogenic Δ5*ppi* and Δ6*ppi* derivative were adjusted to an optical density OD_600_ 0.1 and serially spot diluted on M9 minimal medium and LA medium. Data presented are from one of the representative experiments.

*E. coli* genome contains six genes, whose products represent all three known families of folding catalysts with the peptidyl-prolyl *cis/trans* isomerase activity in the cytoplasm. To gain insights about their function, strains with a single non-polar gene disruption were constructed and transduced into commonly used *E. coli* K-12 strain BW25113 and tested for any perceivable defects in the colony size, motility, cellular morphology and sensitivity towards antibiotics or factors that cause defects in protein folding and growth properties at different temperatures. Individually all the genes were found to be dispensable; however, Δ*fkpB* derivatives exhibited significant growth defects reminiscent of phenotypes ascribed to *lytB* (*ispH*) mutants [13] such as hypersensitivity to ampicillin (1.5 μg/mL) and to ethanol (4%). Sequence examination of *fkpB* and *ispH* coding regions revealed that their transcription and translation is coupled. Thus, a new non-polar deletion of the *fkpB* gene was constructed and such a (Δ*fkpB ispH*^C^) strain exhibited growth similar to the wild type and was further used to construct strains lacking all six cytoplasmic PPIs (for simplicity referred as Δ6*ppi*) using bacteriophage P1-mediated transductions. All transductions were carried in parallel on M9 minimal and Luria Agar (LA) rich medium at 30 and 37 °C. Strains lacking five out of six PPIs exhibited a reduction in the colony size (Figure 1). Hence, to obtain a six-deletion strain and assess any essentiality, Δ5*ppi* strains were transformed with either the vector alone or the plasmid carrying the wild-type copy of gene to be deleted. When Δ5*ppi* derivatives were used as recipients, viable transductants were obtained on M9 medium at 30 °C as well as 37 °C, but not on LA medium when the vector alone was present (Table 1). For further studies, we used a Δ6*ppi* derivative in BW25113 (SR18292) without the vector after verification by PCR the absence of all six *ppi* genes. In the absence of plasmid vector, a Δ6*ppi* derivative forms small colonies on LA at 37 °C with a reduction of colony forming ability by 10^3^ as compared to the wild type, but is not viable at either 43 or 23 °C (Table 1, Figure 1). Further, such bacteria were unable to grow even at 30 or 37 °C when glucose was replaced by glycerol as the carbon source. Overall, these results lead us to conclude that the PPIase activity is essential for optimal growth.

### 2.2. Protein Folding Defects—Accumulation of Various Proteins in Aggregation Fractions in Δ6ppi Bacteria

To investigate the cellular function of cytoplasmic PPIs in protein folding, protein folding defects were examined in the Δ6*ppi* strain SR18292 in comparison to its parental wild-type strain BW25113. Exponentially grown cultures in M9 minimal medium at 37 °C (permissive growth conditions) were washed and shifted to Luria-Bertani (LB) medium at 30 °C for 2 h (conditions poorly tolerated by Δ6*ppi* bacteria). Protein aggregates were obtained after careful removal of membrane proteins using previously established procedure [14]. At 30 °C, as expected, wild-type bacteria did not show any significant amount of protein aggregates. However, under the same conditions, extracts from the Δ6*ppi* strain showed a significant increase in the accumulation of protein aggregates accounting for more than 10% of total proteins. Proteins that aggregated in the Δ6*ppi* strain were resolved by SDS polyacrylamide gel electrophoresis (SDS-PAGE) and analyzed by 2-dimensional electrophoresis. The identity of major protein spots, corresponding to aggregation-prone proteins present in the Δ6*ppi* strain and absent in the parental wild type, was obtained by MALDI-TOF (Table S1). Several proteins that were found to aggregate include major subunits of RNA polymerase and associated factors (RpoB, RpoC, RpoA, Rho), several proteins involved in DNA replication and repair (DnaE, RecB, GyrA, RecA, RuvB, SeqA, LexA), RNA degradation pathway-related proteins (HrpA, CsrD, RhlB, DeaD, Eno), cell division proteins (FtsZ, ZapD, FtsX), those involved in the protein translation (TufA, TufB, InfB, PrfC), proteins required in various metabolic pathways and sugar uptake, transcription factors (Crl, Lrp, Crp, NarL, ArcA, OmpR, ModE), tRNA modifying enzymes, some proteins needed in early steps of lipid A biosynthesis and fatty acid metabolism (LpxA, LpxC, FabA, FabB, FabZ), protein folding and degradation machinery components (IbpB, HslU, ClpX, FtsH), proteins related to cellular redox homeostasis such as alkyl hydroperoxide reductase subunits (AhpC, AhpF), osmotic stress-related proteins (OtsA, BetB, ProP), many ribosomal proteins, including S2, and proteins such as Der, which are required for the ribosomal stability/integrity (Table S1). Interestingly, several of proteins that aggregate in Δ6*ppi* bacteria are essential for the bacterial viability and can help to explain the collective essentiality of cytoplasmic PPIs for the viability under standard laboratory growth conditions. Proteins that were found to aggregate in Δ6*ppi* bacteria were analyzed using the available protein database (PDB server) revealing that some of them indeed contain either known or predicted *cis* proline residues, while many are proline rich suggesting a requirement of PPIs in the catalysis of proline isomerization (Table S1).

### 2.3. The PpiB Protein Is the Major Contributor of PPIase Activity in the Cytoplasm

**Figure 2:**
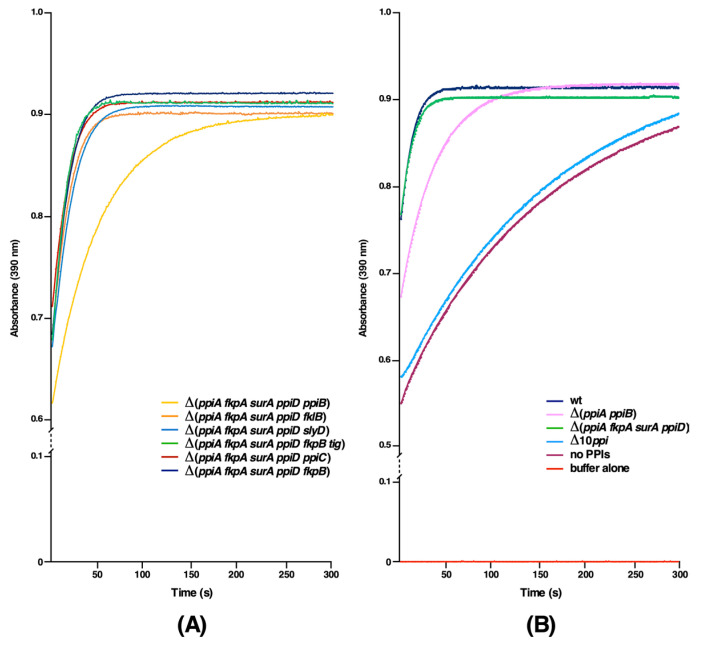
PpiB is the major contributor of PPIase activity in the cytoplasm. (**A**) The PPIase activity in soluble extracts obtained from six sets of strains lacking periplasmic PPIs and one of the cytoplasmic PPI were used to measure the PPIase activity, revealing that the absence of PpiB leads to highly reduced PPIase activity. In all cases, total protein concentration was 10 mg/mL and N-Suc-Ala-Ala-cis-Pro-Phe-p-nitroanilide was used as the substrate. The PPIase activity was measured in a chymotrypsin-coupled assay. (**B**) Under similar conditions, extracts from the wild type, Δ(*ppiA ppiB*), Δ(*ppiA fkpA surA ppiD*), Δ10*ppi* strain lacking all PPIs were used to measure the PPIase activity.

Up to now, the relative contribution of each cytoplasmic PPI to the total enzymatic pool has not been reported. To measure only the PPIase activity of cytoplasmic PPIs, a strain that lacks four periplasmic PPIs was constructed to introduce different null alleles of genes encoding cytoplasmic PPIs to quantify their PPIase activity in a single copy. Measurement of the PPIase activity of soluble cell extracts obtained from such strains revealed that among cytoplasmic PPIs PpiB contributes the bulk of peptidyl-prolyl *cis/trans* isomerase activity (Figure 2A). The relative order of PPIase activity is PpiB > FklB > SlyD > Tig > PpiC > FkpB. Surprisingly, the strain lacking four periplasmic PPIs and the *tig* gene consistently showed the higher PPIase activity than its parental strain. As Tig is an abundant ribosome-associated protein with a chaperone-like activity, suggested that the expression of one or more cytoplasmic PPIs might be up-regulated in a *tig*-deleted strain. Thus, an individual null allele of rest of five cytoplasmic PPI-encoding genes were introduced into a SR20072 derivative lacking the *tig* gene and analyzed for the PPIase activity. Among such strains, the deletion derivative of the *fkpB* gene alone showed the significantly reduced PPIase activity that can be attributed to Tig (Figure 2B). Thus, we can conclude that the observed lack of reduction in the PPIase activity in Δ(*ppiA surA ppiD fkpA tig*) as compared to Δ(*ppiA surA ppiD fkpA*) is due to the increased contribution from FkpB, when the Tig protein is absent, and the major contributor to the cytoplasmic pool of the PPIase activity activity is from the PpiB protein (Figure 2A).

Further, we sought to identify major contributors to the overall pool of total cellular PPIase activity. Thus, several double deletion combinations were constructed and used for the PPIase activity measurement. Among these, the most striking is a drastic reduction in the PPIase activity in Δ(*ppiA ppiB*) (Figure 2B). PpiA located in the periplasm and PpiB in the cytoplasm are highly homologous proteins and both belong to the cyclophilin family. Furthermore, we also constructed strains devoid of all ten PPIs, revealing only the presence of a very little residual PPIase activity (Figure 2B). However, it is pertinent to point out that strains lacking all ten PPIs exhibit severe growth defects with doubling time of 270 min and are unable to reach an OD_600_ above 0.3 at 37 °C. Such a phenotype highlights the importance of this class of folding catalysts for the bacterial viability. Taken together, we can conclude that PpiA and PpiB are the main contributors to the PPIase activity.

### 2.4. Δ6ppi Bacteria Exhibit the Constitutive Induction of RpoH-Regulated Heat Shock Response

**Figure 3:**
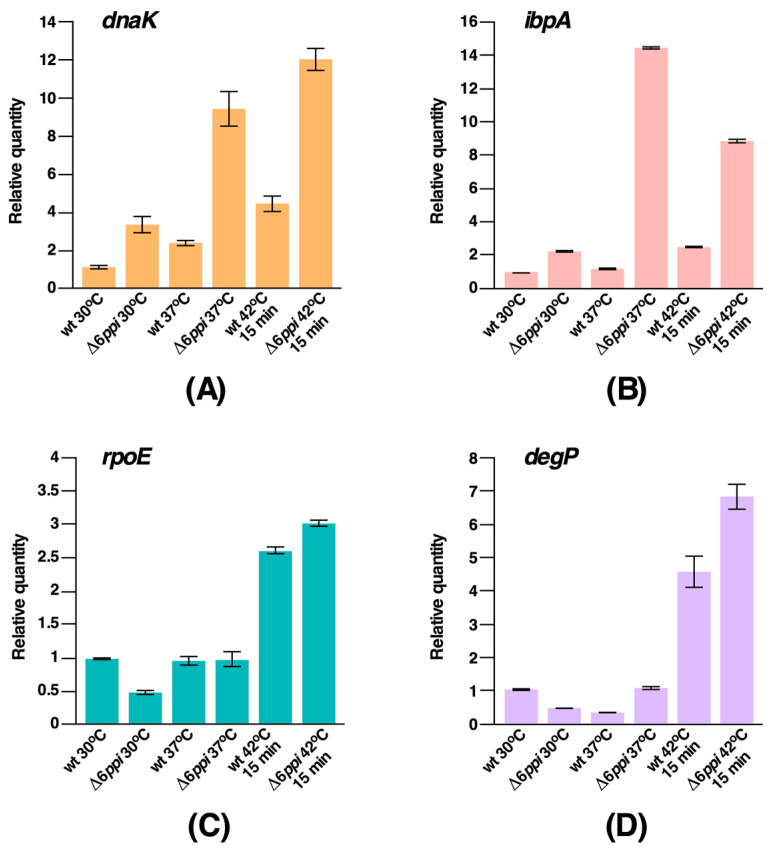
Δ6*ppi* bacteria exhibit constitutive elevated transcription of RpoH-regulated heat shock genes but reduced level of RpoE-transcribed genes at 30 and 37 °C. qRT-PCR analysis of mRNA isolated from the wild type (wt) and its Δ6*ppi* derivative bacteria grown up to an OD_600_ of 0.2 in M9 medium at either 30 or after shift to LB at 37 °C. In parallel, total RNA was extracted after heat shock for 15 min at 42 °C. Quantification of data for the *dnaK* gene (Panel **A**), the *ibpA* gene (Panel **B**), the *rpoE* gene (Panel **C**) and the *degP* gene (Panel **D**).

It is well established that accumulation of misfolded proteins and overall defects in protein folding process induces cellular stress responsive pathways that can mitigate such stresses. *E*. *coli* uses two such stress combative pathways that leads to the induction of heat shock response. As Δ6*ppi* bacteria exhibited gross defects in terms of growth, extensive protein aggregation and also many transcription factors were found enriched during the analysis of proteins that accumulate in aggregation fractions, we analyzed the fate of major stress responsive pathways, such as the RpoH-regulated heat shock response and the RpoE-controlled envelope stress response. Thus, quantitative real-time polymerase chain reaction (qRT-PCR) analysis of well-known RpoH-regulated heat shock genes was performed, using total cellular mRNA isolated with or without temperature upshift. mRNA was also extracted from isogenic wild-type and Δ6*ppi* bacteria after shift from M9 30 °C or 37 °C to LB 37 °C. Data from qRT-PCR of highly conserved heat shock genes *dnaK* and *ibpA* are presented, revealing increased abundance of transcripts of these heat shock genes in Δ6*ppi* bacteria as compared to the wild type even under permissive growth conditions of either 30 or 37 °C (Figure 3A,B). Noteworthy is the hyperinduction of transcription of the *ibpA* heat shock gene at 37 °C. These data are consistent with a role of IbpA in binding to protein aggregates. A 15 min transient shift of culture of Δ6*ppi* bacteria from 30 °C to 42 °C in M9 minimal medium also resulted in the increased expression of all major heat shock genes in the wild type as well as in Δ6*ppi* bacteria (Figure 3). Thus, these data show that in the absence of PPIs heat shock response under the control of the *rpoH* gene is constitutively induced under permissive growth conditions in Δ6*ppi* strains and they exhibit an elevated induction of transcription of heat shock genes after temperature shift to 42 °C.

Concerning the RpoE-regulated stress response, the basal level transcriptional activity of the *rpoE* gene, which regulates the envelope stress response, is reduced in Δ6*ppi* bacteria at 30 °C and is unaltered at 37 °C. Regarding the *degP* gene, whose transcription is subjected to dual control by RpoE and CpxR/A systems [15], its transcriptional activity is also reduced at 30 °C, although its transcription at 37 °C remains unaltered (Figure 3C,D), which is in contrast to the constitutively enhanced activity of RpoH-dependent heat shock regulon. However, a 15 min shift to 42 °C causes the induction of transcription of the *rpoE* as well as the *degP* genes in Δ6*ppi*, which is comparable with their induction in the parental wild-type strain. Thus, we can conclude that while the RpoH-regulated heat shock response is constitutively induced in Δ6*ppi* bacteria even under permissive growth conditions, the RpoE-regulated transcription under similar growth conditions is rather dampened.

### 2.5. Identification of Substrates of the PpiC Protein Reveals a Role in Oxidative Stress, Transcriptional and Essential Metabolic Processes

**Figure 4:**
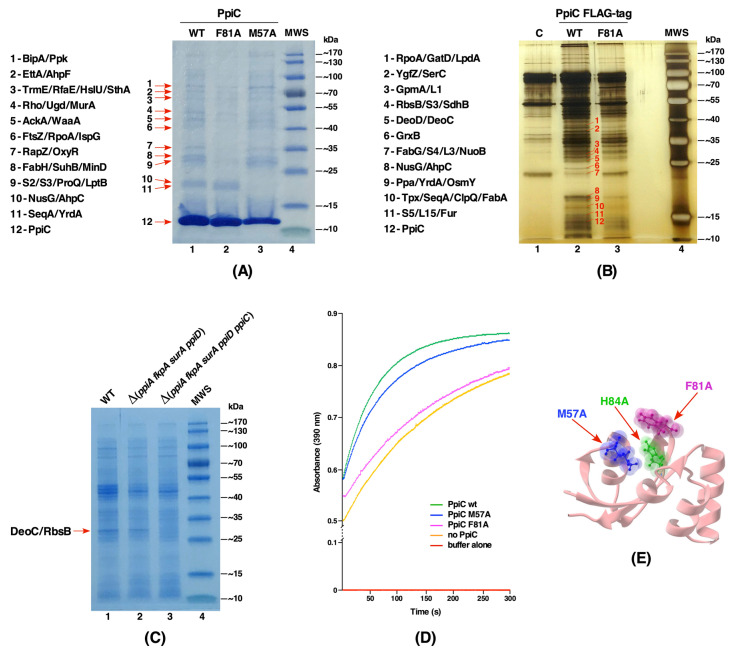
(**A**) Purification profile of wild-type PpiC, PpiC F81A and PpiC M57A after the induction in a Δ6*ppi* derivative with 100 μM IPTG. Proteins were resolved on a 12.5% SDS-PAGE. Co-eluting proteins are indicated by arrows with their identity marked. MWS indicates the molecular weight standard (Thermo Scientific). (**B**) Proteins identified after immunoprecipitation with an anti-FLAG M2 monoclonal antibody of extracts from the wild-type strain without FLAG tag as a control (lane marked C) and strains with a chromosomal FLAG-tagged PpiC wt and PpiC F81. Immunoprecipitated proteins are indicated. (**C**) Soluble proteins from equivalent amounts of cells from the wild type (wt), its isogenic strain derivatives lacking four periplasmic PPIs and its derivative strain additionally lacking PpiC, were resolved on a 12.5% SDS-PAGE. Missing proteins in the absence of Δ*ppiC* derivative are indicated by arrow. (**D**) The PPIase activity in the chymotrypsin-coupled assay with 0.5 μM purified wt PpiC and its mutants with either M57A or F81A are plotted. Controls without enzyme and buffer are also plotted. (**E**) Position of M57, F81 and H84 residues in the structure of PpiC (PDB 1JNT).

Up to now, no systematic study has been carried out to identify substrates of individual cytoplasmic PPIs. Multipronged strategies were employed to identify in vivo substrates of PpiC. Firstly, the wild-type *ppiC* gene and its variants with single amino acid substitutions in residues predicted to correspond to either the potential active site or the substrate-binding site based on the available solution structure were cloned in the T7 polymerase-based expression plasmid pET24b. We chose highly conserved amino acids Met57 and Phe81 and replaced them with Ala, since they are predicted to form a part of substrate-binding and catalytic domains of PpiC, respectively [11]. In each plasmid construct, the coding region was appended in-frame with a C-terminal cleavable Hexa-His tag and used to transform SR21984 lacking all six PPIs to prevent the presence of other PPIs during purification. After the induction with IPTG (100 μM), proteins were purified using linear gradient of imidazole in Ni-NTA affinity chromatography and eluting protein fractions resolved by SDS-PAGE. The identity of co-eluting proteins with His-tagged PPIs in such pull-down experiments was revealed by MALDI-TOF. Comparison of co-elution profiles of the wild-type Hexa-His-tagged PpiC and Hexa-His-tagged PpiC mutant derivatives revealed that most prominent substrates of PpiC include the BipA GTPase, the translational factor EttA, YrdA, FabH, RbsB, transcription-related proteins (NusG, SuhB, Rho, RpoA), the RNA chaperone ProQ, the GlmY/GlmS sRNA-binding protein RapZ, OxyR, RpsB, RbsC (S2/S3) ribosomal proteins. Other substrates identified include the polyP kinase Ppk, the essential lipid A transporter LptB and AhpC/AhpF subunits of alkyl hydroperoxide reductase. All these co-eluting proteins identified with the wild-type PpiC were either absent or with highly reduced amounts in PpiC F81A and PpiC M57A variants in such pull-down experiments (Figure 4A). Most prominent differences in the co-elution profile are observed when proteins from pull-down experiments from the wild-type PpiC vs. PpiC M57A are compared. This comparison reveals the absence of NusG, YrdA and AhpC proteins in M57A variant (Figure 4A).

In a complementary and more sensitive second approach, the wild-type chromosomal copy of the *ppiC* gene was epitope-tagged with 3xFLAG using recombineering. In parallel, PpiC F81A mutation was introduced and thus an isogenic strain with an appended in-frame C-terminal 3xFLAG epitope with a chromosomal mutation in the putative catalytic site was generated. Total cell extracts from such FLAG-tagged wild-type PpiC and its mutant F81A were immunoprecipitated with an anti-FLAG M2 monoclonal antibody and bound substrates after elution were revealed by silver-staining after SDS-PAGE. This analysis revealed common substrates that were identified as co-eluting proteins in pull-down studies like RpoA, AhpC, YrdA, SeqA, NusG and RpsC, validating them as clients of PpiC (Figure 4B). Interestingly, many substrates like GrxB, AhpC, YrdA, NusG, and SeqA were not immunoprecipitated when extracts from the strain carrying PpiC F81A::3xFLAG were used, consistent with pull-down experiments as compared to their presence in immunoprecipitated extracts from the wild-type FLAG-tagged PpiC (Figure 4A,B). Some additional substrates, revealed with immunoprecipitations in the case of wild-type PpiC::3xFLAG, were proteins such as GrxB, Ppa, GpmA, YgfZ, DeoC, and RbsB, which were missing in pull-down experiments. As immunoprecipitations were performed with cell extracts from FLAG-tagged strains carrying a single-copy wild-type and its F81A derivative, without any overexpression, such results are physiologically more relevant than using overexpressing systems. Due to limitations of current recombineering techniques, a chromosomal M57A mutation in a FLAG-tag system could not be constructed. Finally, in the third approach, we compared the profile of total soluble proteins obtained from isogenic strains lacking all periplasmic PPIs Δ(*surA ppiD ppiA fkpA*) (SR20072) and its derivative SR20098 Δ(*surA ppiD ppiA fkpA ppiC*) additionally lacking the *ppiC* gene (Figure 4C). MALDI-TOF analysis of missing protein(s) in SR20098 identified DeoC and RbsB to be absent when the Δ*ppiC* mutation was introduced (Figure 4C). These results further reinforce their identification in either pull-down or immunoprecipitation studies, validating them as PpiC substrates.

To correlate the identification of substrates and gain better insight in the function of PpiC in relationship with its PPIase activity and relevance of substrate-binding site, purified wild-type PpiC, PpiC M57A and PpiC F81A proteins were obtained after rifampicin treatment and used to measure their activity in chymotrypsin-coupled assay. Results from these measurements revealed that PpiC F81A mutant protein has lost its PPIase activity, while PpiC M57A still retains a substantial PPIase activity (Figure 4D). Based on the structural analysis, Met57 is located in the putative substrate- binding domain of PpiC [11] (Figure 4E) and thus helps to explain the inability to recognize some of the substrates as revealed by pull-down studies. Similarly, the absence of several substrates, when PpiC F81A::3xFLAG was used in immunoprecipitations, implies a dependence of several substrates, such as GrxB, AhpC, NusG (Figure 4) on the PPIase activity of PpiC. Thus, we can conclude that both substrate-binding and PPIase active site residues are required for PpiC’s function.

### 2.6. Substrates of FkpB Include the RpoE Sigma Factor, Proteins Related to Cell Shape/Division and Lipopolysaccharide (LPS) Transport

**Figure 5:**
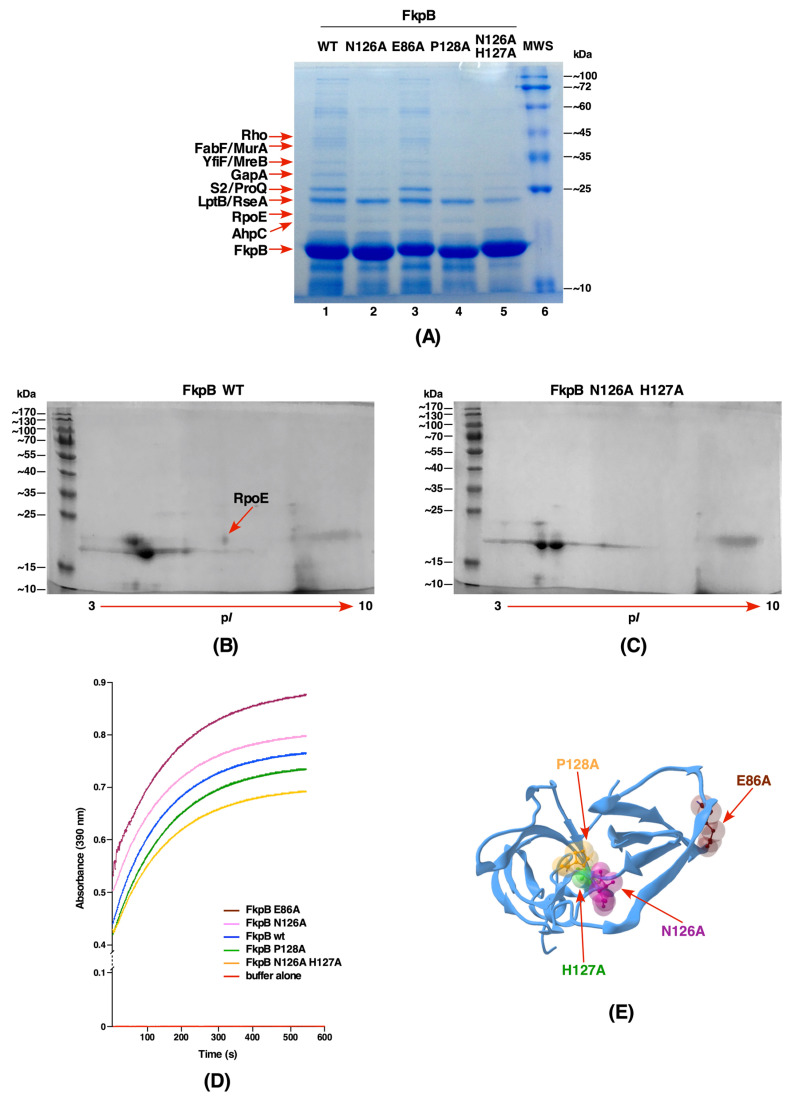
The RpoE sigma factor is one of the substrates of FkpB. (**A**) Co-purification profile of the wild-type FkpB protein and its derivatives N126A, P128A, E86A and with N126A H127 double mutation after the induction in a Δ6*ppi* derivative with 100 μM IPTG. Proteins were resolved on a 12.5% SDS-PAGE and co-eluting proteins are indicated and their identity shown. (**B**) Picture of dried 2-D gels of purified FkpB and co-eluting RpoE. The identity of the spot corresponding to RpoE was obtained from MALDI-TOF and is indicated. (**C**) 2-D gel of purified FkpB variant N126A H127. (**D**) The PPIase activity in the chymotrypsin-coupled assay with 0.5 μM purified of above mentioned FkpB variants in comparison to the wild type is plotted. (**E**) Position of various FkpB residues, which were mutated, is indicated in the structure of FkpB (PDB 4DT4).

The FkpB PPIase of *E. coli* exhibits structural similarity with the SlyD protein [8,12]. However, its cellular substrates and physiological function remains to be elucidated. Thus, as with PpiC, we identified substrates using pull-down experiments with FkpB. To achieve this, the minimal coding region of the *fkpB* gene with a C-terminal in-frame Hexa-His tag was cloned in the T7 polymerase-based expression plasmid pET24b and protein expression induced in the Δ6*ppi* derivative as described above. In the case of FkpB, some of the prominent co-eluting proteins using pull-down experiments are: RpoE (envelope stress response regulator sigma E), LptB (cytoplasmic ATPase component of LPS ABC transport complex), the RseA anti-sigma factor specific to RpoE, RpsB and RpsC (S2 and S3, respectively) ribosomal proteins, the RNA chaperone ProQ, Rho transcriptional terminator factor, the GapA protein which is involved in glycogenesis, putative methyl transferase YfiF related to RNA processing, the cell shape determining protein MreB, an alkyl hydroperoxide reductase subunit C AhpC and also some proteins that are involved in the phospholipid/fatty acid synthesis such as FabF. To gain insights in the structure-function analysis of FkpB, single amino acid substitutions include E86A, N126A, P128A, and a H126A N127A double mutant were constructed on plasmids. Such Hexa-His-tagged FkpB mutant proteins were purified and the profile of co-eluting proteins were analyzed in comparison with co-eluted proteins obtained from the wild-type His-tagged FkpB (Figure 5A). All the mutated residues were selected on the basis of their location in the putative active site of FkpB (Figure 5) [12]. Examination of co-eluting proteins with substitution of E86A in FkpB yielded a profile quite similar to that of the wild-type. However, quite importantly the co-elution of RpoE was diminished in FkpB N127A, P128A variants and abolished in FkpB N126A H127A double mutant. These results were further validated by analysis of co-eluting proteins upon two-dimensional (2D) gel electrophoresis. As is evident, the protein spot corresponding to RpoE was visualized and its identity established by MALDI-TOF analysis using co-eluted proteins from His-tagged wild-type FkpB (Figure 5B). However, the protein spot corresponding to RpoE is missing in the 2D gel when the co-eluted proteins from His-tagged FkpB H126A N127A double mutant were applied (Figure 5C). It is worth noting an overlap of substrates between PpiC and FkpB for ribosomal proteins S2 and S3, and AhpC, LptB, Rho, and ProQ which are also common substrates. Some of these substrates were validated by in vitro binding assays (see below).

Next, we measured the PPIase activity of wild-type FkpB and its variants that were tested in pull-down studies using the standard chymotrypsin-coupled assay. This analysis revealed a significantly reduced PPIase activity of FkpB H126A N127A double mutant protein as compared to the wild-type (Figure 5D,E). Furthermore, FkpB P128A mutant protein was also found to be defective in the catalysis of the prolyl isomerization, while E86A variant still retains the PPIase activity although reduced as compared to the wild type (Figure 5D,E). These results are consistent with a predicted role of H126, N127, and P128 residues in the catalysis of prolyl isomerization.

### 2.7. The PpiB Protein Has Larger Number of Substrates That Require Its PPIase Activity

**Figure 6:**
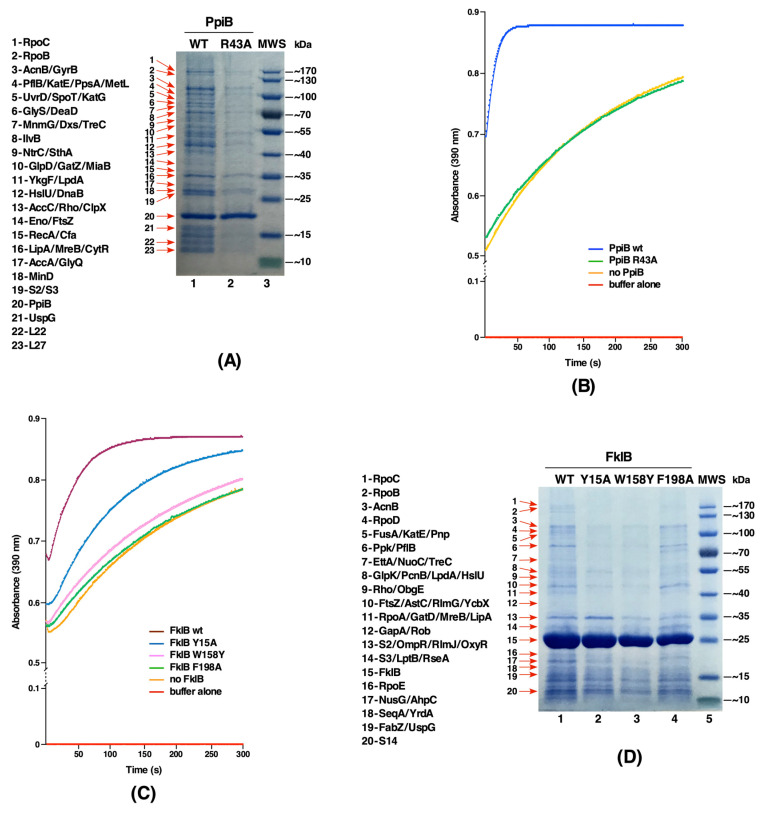
Identification of substrates of PpiB and FklB. (**A**) Co-purification profile of wild-type PpiB and PpiB R43A proteins. Co-eluting proteins present in the wild-type that are either absent or with reduced abundance are indicated. (**B**) The PPIase activity in the chymotrypsin-coupled assay with purified wt PpiB and PpiB R43A (0.5 μM each) is indicated. Controls without enzyme and buffer are also plotted. (**C**) The PPIase activity in the chymotrypsin-coupled assay with 0.5 μM purified FklB variants in comparison to the wild-type FklB, without enzyme and buffer alone are plotted. (**D**) Co-purification profile of wild-type FklB, and its derivatives Y15A, W158Y and E86A. Proteins were resolved on a 12.5% SDS-PAGE and co-eluting proteins are indicated and their identity shown.

The PpiB protein was the first cytoplasmic *E. coli* PPIase identified and biochemically its activity elucidated [16,17]. As shown in this work, PpiB is the major source of PPIase activity in the cytoplasm of *E*. *coli*. However, little information is available concerning its cellular substrates. Thus, in this study we cloned the wild-type and putative active site variant of the *ppiB* gene with appended His-tag at the C-terminal end of its coding sequence and expressed from the T7 promoter-based expression vector pET24b. Pull-down experiments with His-tagged PpiB revealed largest number of co-eluting polypeptides as compared to similar experiments with other PPIs (Figure 6A). While the identity of several proteins could be obtained, we focused more on those proteins which do not co-purify with PpiB R43A variant. PpiB R43A substitution was constructed, since its counterpart R55 residue in human Cyp18 is known to have a very low PPIase activity [18]. Examination of purification profile of PpiB R43A variant revealed that the vast majority of co-eluting proteins observed in the case of wild-type His_6_-tagged PpiB were either absent or their abundance is reduced (Figure 6A). Some of the co-purifying proteins observed with the wild-type PpiB, but with a reduced abundance with His_6_-tagged PpiB R43A, are: components of RNA polymerase β and β’ subunits, transcription factors Rho, CytR and NtrC, tRNA modification enzymes MiaB, MnmG, the RNA helicase DeaD, Eno, which is a part of RNA degradosome, catalase subunits HPI and HPII (KatE and KatG, respectively), enzymes related to DNA replication/recombination like GyrB, RecA, several proteins related to energy metabolism such as AcnB, GlpD, LpdA, and SthA, components of biotin-carboxyl carrier protein assembly which are also involved in the fatty acid synthesis like AccA and AccC, stress-related proteins like ClpX, HslU and UspG (Figure 6A). Additionally, in pull-down studies proteins involved in cell division/cell shape (FtsZ, MreB and MinD) were observed as well as ribosomal proteins like RpsB/C (S2 and S3 proteins) and RplV (Figure 6A). Thus, PpiB substrates are quite broad in function and most of these proteins were only identified during purification of the wild-type PpiB but not with PpiB R43A.

Next, we verified if indeed PpiB R43A mutant protein is defective in its PPIase activity. As shown, this mutation nearly abolished the PPIase activity of PpiB (Figure 6B), consistent with observations with corresponding Cyp18 R55 variant. Taken together, we can conclude that most of the substrates identified in these studies require PpiB’s PPIase activity for their interaction.

### 2.8. Identification of Active Site Residues of the FklB Protein and Identification of its Substrates Reveal Certain Unique and Some with an Overlap with Other PPIs

No substrate of FklB has been reported thus far in *E*. *coli* and its biochemical studies are limited, although it has been shown to be a dimeric protein [19]. To investigate the function of FklB, experiments were undertaken to identify its binding partners and determine the importance of highly conserved amino acid residues predicted to impact its PPIase and substrate-binding activity. Hence, we generated single amino acid substitutions based on homology modeling with FKBP22 of *Shewanella* sp. [20]. Homology data suggest *E*. *coli* FklB to be a V-shaped dimer, with the N-terminal domain required for dimerization and the C-terminal domain to constitute the catalytic domain. Thus, mutations in FklB with Y15A, W158Y and F198A substitutions were generated and cloned in pET28b expression vector. Such *fklB* mutants and the wild type were expressed in the Δ6*ppi* derivative SR21984 and proteins were purified under identical conditions. Substitutions like Y15A in the N-terminal domain should render FklB in the monomeric state [20,21], while substitutions in C-terminal residues W158 and F198 can affect the PPIase and the chaperone-like activity. Measurement of the PPIase activity using *N*-Suc-Ala-Ala-*cis*-Pro-Phe-*p*-nitroanilide peptide as a substrate revealed a severe reduction of PPIase activity in the case of FklB W158Y and a loss of PPIase activity with FklB F198A mutants (Figure 6C). However, Y15A mutation in the N-domain retained the PPIase activity, although it is reduced as compared to the wild type. In parallel, pull-down experiments revealed a substantial reduction in the number of co-eluting proteins, when FklB Y15A and W158Y variants were subjected to affinity purification (Figure 6D). However, when FklB F198A was purified, many proteins that co-elute with the wild-type Hexa-His-tagged FklB were also observed, although certain proteins were reduced in abundance. These results suggest that both the dimeric nature and the PPIase activity are required for the FklB function. However, all substrates may not require FklB’s PPIase activity.

Identification of potential substrates of FklB from co-eluting proteins revealed some unique interacting partners like the transcriptional regulator Rob, which is involved in regulation of antibiotic resistance and stress response [22,23], the transcriptional factor Crl regulating RpoS expression/activity, *ompF*/*C* transcriptional regulator OmpR, the plasmid copy number regulator PcnB, proteins related to translational process like the ribosome assembly-related GTP-binding protein ObgE, ribosomal proteins and RlmG/RlmJ involved in methylation of 23S rRNA (Figure 6D). However, many co-purifying proteins were common with FkpB, PpiC and PpiB. These include proteins like AhpC, OxyR, TreC, NusG, Rho, RpoA, and RpoE. However, higher overlap is observed with FkpB, suggesting redundancy in substrate usage by different cytoplasmic PPIs.

### 2.9. Validation of RpoE, RseA, and S2 Proteins as Client Proteins of FkpB

Based on pull-down experiments, several proteins were identified as potential substrates of FkpB. These include the S2 ribosomal protein, the RpoE sigma factor and the anti-sigma factor RseA. The RpoE sigma factor is an alternative sigma factor that in the complex with RNA polymerase initiates transcription of several genes whose products are involved in either folding and assembly of various components of the OM including major OMPs and lipopolysaccharide or transcription of sRNAs that negatively regulate the synthesis of OMPs and the abundant envelope murein lipoprotein Lpp (reviewed in [24]). RseA acts as the anti-sigma factor for RpoE and regulates the RpoE availability in response to envelope stress [25,26].

**Figure 7:**
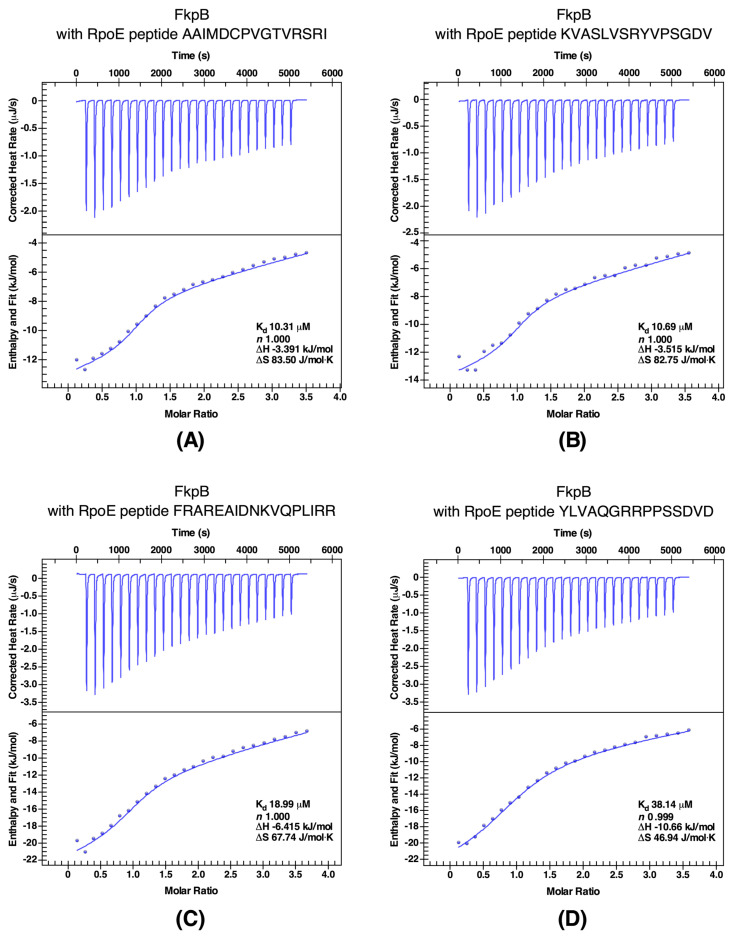
Quantification of binding affinity of FkpB with four different peptides derived from RpoE (Panel **A**–**D**). FkpB was titrated into peptides indicated on the top of each panel in ITC cell. Raw data (top half of each panel) and integrated heat measurements enthalpy (bottom part) are shown. The calculated stoichiometry (*n*) and dissociation constant (*K*_d_) are indicated in insets.

To further investigate the interaction between RpoE and RseA with FkpB, binding affinity measurements were performed with isothermal titration calorimetry (ITC) using various peptides spanning different segments of RpoE and RseA. The best binders were peptides P2 (KVASLVSRYVPSGDV) and P4 (AAIMDCPVGTVRSRI) that were derived from the most conserved regions 2 and 4 of RpoE. The titration of FkpB into peptides P4 and P2 was exothermic (Figure 7A,B). The binding curves were fit to a single binding-site mode (*n* = 1), revealing a binding affinity with a *K*_d_ of 10.69 μM for P2 peptide and with a *K*_d_ of 10.31 μM for P4 peptide (Figure 7A,B). Another peptide designated P3 (FRAREAIDNKVQPLIRR) again covering the conserved region 4 that includes amino acid like R178 shown previously critical for RpoE and RseA interaction and the RpoE activity [15,27], when titrated with FkpB also revealed a high binding affinity with a *K*_d_ of 18.99 μM (Figure 7C). Similarly, fitting the data to a single-site binding model (*n* = 1) with peptide P1 (YLVAQGRRPPSSDVD) corresponding to the C-terminal end of conserved region 2.4 of RpoE, also yielded thermodynamically favorable interaction, although with a relatively modest binding affinity with a *K*_d_ of 38.14 μM (Figure 7D). Taken together, ITC measurements of affinities between RpoE-base peptides with FkpB are driven by a favorable change in enthalpy.

**Figure 8:**
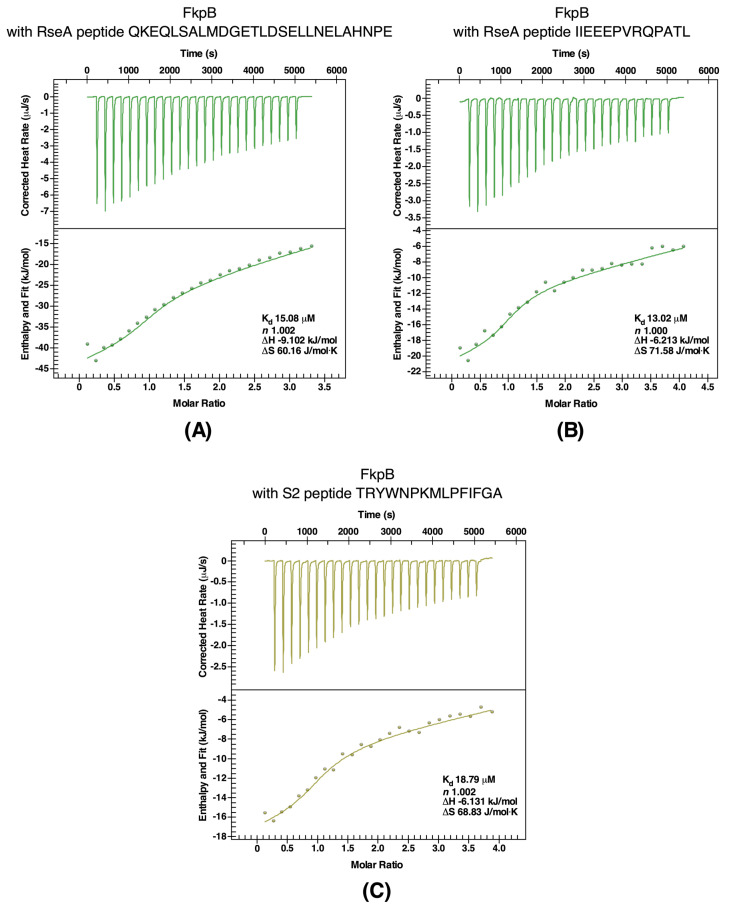
Quantification of binding affinity of FkpB with peptides from RseA (Panel **A**,**B**) and the peptide from the S2 ribosomal protein (Panel **C**). Heat rates and fit for ITC experiment, where FkpB is injected into above-mentioned peptides. The calculated stoichiometry (*n*) and dissociation constant (*K*_d_) are indicated in insets.

Since the anti-sigma factor RseA also co-elutes with FkpB, binding affinities were measured using ITC with peptides covering the most conserved regions in the N-terminal domain of RseA. Thermograms of two best binders are presented (Figure 8B,C). Peptide Rse1 corresponding to amino acid 2-29 of N-RseA binds to FkpB with a *K*_d_ of 15.08 μM and with a stoichiometric value (*n*) of 1.002 (Figure 8B). Similarly, a high affinity binding with a *K*_d_ of 13.02 μM was observed with peptide Rse3 (IIEEEPVRQPATL) (Figure 8C). Furthermore, interaction of peptides Rse1 and Rse3 with FkpB were exothermic and driven by favorable change in enthalpy with ΔH being −9.102 and −6.213 kJ/mol, respectively.

As shown in the analysis of accumulation of proteins that tend to aggregate in a Δ6*ppi* mutant strain even under permissive growth conditions, the S2 ribosomal protein (RpsB) was enriched in aggregation fractions (Table S1). Furthermore, the S2 ribosomal protein is one of the prominent proteins that co-elutes with FkpB. The S2 ribosomal protein was also in earlier independent studies identified as a potential substrate of FkpB and a peptide containing a *trans* Pro residue derived from the S2 ribosomal protein was used in such studies to show favorable interaction with FkpB [12]. Thus, we validated binding of the same S2-derived peptide using ITC. Measurement of binding affinity by ITC to this peptide gave a binding affinity with a *K*_d_ of 18.79 μM (Figure 8A), which is similar to that reported earlier 12], thus reinforcing our results obtained with pull-down experiments. Thus, taken together, ITC experiments validated the RpoE sigma factor, the anti-sigma factor RseA and the ribosomal protein S2 as binding partners of FkpB PPIase. These results also draw support from co-purification of these client proteins with FkpB.

### 2.10. AhpC as a Client Protein of PpiC and FkpB

**Figure 9:**
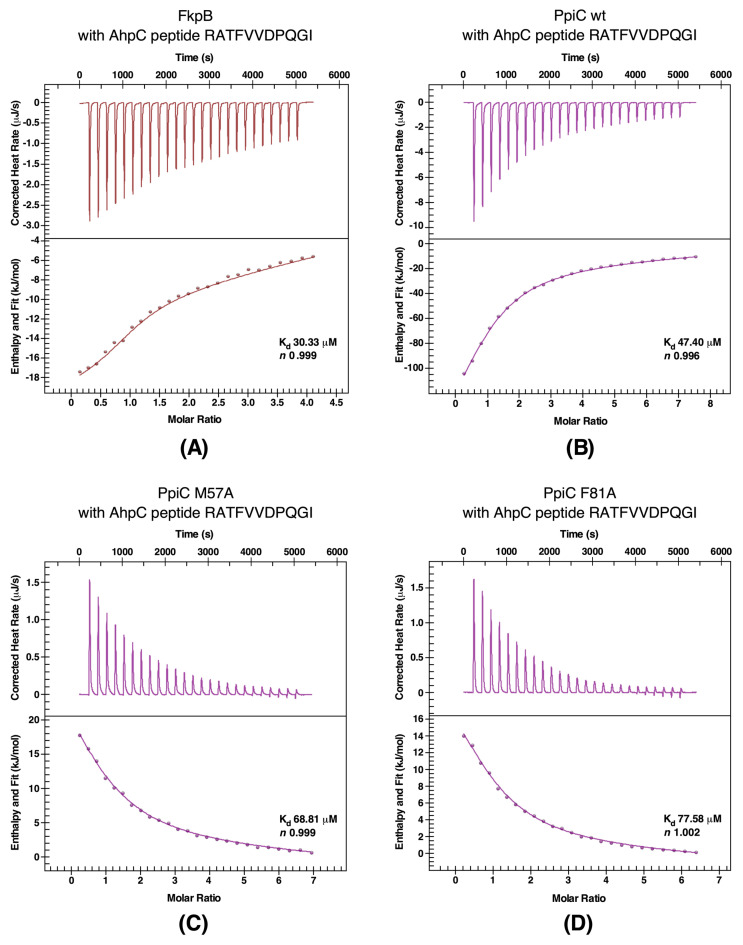
ITC experiments with the AhpC-derived peptide and FkpB (Panel **A**) and PpiC wild-type (Panel **B**) and PpiC M57A (Panel **C**) and PpiC F81 (Panel **D**). Heat rates and fit for ITC experiment, and the calculated stoichiometry (*n*) and dissociation constant (*K*_d_) are indicated in insets.

As shown above, AhpC protein was identified as one of the aggregation-prone proteins in Δ6*ppi* bacteria and as a potential partner of PPIs, since AhpC was also found to co-purify with PpiC and FkpB. Such purified proteins were used to study their interaction with a peptide from AhpC (RATFVVDPQGI) using ITC. This peptide was chosen, since it is present in one of three segments of AhpC as a potential PpiC binder [11]. Measurement of binding constants revealed that wild-type PpiC and FkpB proteins bind with stoichiometric value (*n*) of 0.99 in each case, indicating that one molecule of these catalysts binds to the AhpC peptide with high affinity (*K*_d_ values of 30 μM and 47 μM, respectively) validating AhpC as their substrate (Figure 9A,B). Reactions in both cases were again exothermic with wild-type PpiC and FkpB with favorable changes in enthalpy.

Since our PPIase measurements established PpiC F81A to have lost its PPIase activity and in immunoprecipitation experiment PpiC F81A::3xFLAG could not show interaction with several substrates identified in pull-down experiments including AhpC, we tested interaction by ITC with the AhpC peptide used in above studies. In parallel, the interaction between another mutant PpiC M57V with the AhpC peptide was analyzed. This analysis revealed that unlike exothermic reaction upon the interaction of wild-type PpiC with the AhpC peptide, the titration of PpiC mutants defective in the PPIase activity and substrate binding into the AhpC peptide yielded endothermic reactions (Figure 9C,D respectively) with unfavorable thermodynamic changes. Thus, comparison of thermodynamic parameters and nature of only exothermic reaction with the wild-type PpiC but not with PpiC mutants, leads us to conclude that defects in the PPIase activity (PpiC F81A) and substrate binding (PpiC M57A) disrupts interaction between AhpC and PpiC. Hence, the PPIase activity of PpiC is required for interaction with AhpC, which is also supported by immunoprecipitation experiments.

## 3. Discussion

It is well established that the kinetics of the Xaa-Pro bond isomerization is intrinsically slow due to the partial double bond nature of the peptide bond [28] and hence is a rate-limiting step in protein folding. Peptidyl-prolyl *cis*/*trans* isomerases (PPIs) constitute a unique ubiquitous group of universally conserved protein folding catalysts that accelerate the catalysis of this rate-limiting step of isomerization around Xaa-Pro bonds. However, in *E. coli* their physiological requirement, the identification of interacting partners and which substrates require their PPIase activity has not been addressed. This is partly due to: (i) the transient nature of PPI-substrate interactions, (ii) the overlap in substrates that masks the phenotype of individual *ppi* deletion, (iii) the absence of systematic studies that address the individual contribution of any PPI to overall enzymatic activity, since no strains have been reported that lack all six PPIs, (iv) the polar effect of gene disruptions in some PPIs-encoding genes on downstream essential gene(s) like the polarity effect of gene disruption of *fkpB* and *ppiB* genes on *ispH* and *lpxH* genes, respectively. Thus, in this work, we for the first time report the construction and characterization of strains that lack all six cytoplasmic PPIs. All deletion derivatives were non-polar and transduced into the wild-type *E*. *coli* strain BW25113. Such Δ6*ppi* deletion derivatives were found to exhibit highly pleiotropic phenotypes. These include the inability to grow under fast growing conditions of rich medium and severe defects in protein folding as manifested by the accumulation of protein aggregates. Examination of protein that aggregate in Δ6*ppi* bacteria revealed that more than 10% cytoplasmic proteins accumulated in the aggregation state. Among various proteins that were identified to aggregate in Δ6*ppi* bacteria, some of them are involved in transcription and RNA processing, translational process, many ribosomal proteins, DNA synthesis/repair/replication enzymes, oxidative stress-related proteins and enzymes that play a key role in central metabolism like TCA cycle, glycerol uptake and its catabolic process and ubiquinone biosynthesis. Consistent with these results, Δ6*ppi* strains cannot utilize glycerol as the sole carbon source. Other aggregation-prone proteins are involved in the assembly and biosynthesis of two essential components of cell envelope: LPS and phospholipids. Synthesis of these components in the cell is tightly regulated and coupled [14] and can contribute to the collective essentiality of PPIs. Although a diverse set of proteins were found to aggregate, yet one can conclude that major defects in Δ6*ppi* bacteria can be attributed to aggregation of proteins related to translation, transcription, maintenance of cell envelope homeostasis and factors related to combating oxidative stress and DNA repair processes. Indeed, during our ongoing studies, we have found that Δ6*ppi* bacteria are super sensitive to oxidative stress, factors that interfere with protein translation/synthesis and protein folding process, variety of DNA and cell envelope damaging agents. Some of these aggregation-prone proteins related to oxidative stress like AhpC and AhpF or ribosomal proteins were indeed again identified during pull-down studies and ITC experiments as bona fide substrates of PPIs. A vast number of proteins that were shown to aggregate in Δ6*ppi* bacteria have also been reported to aggregate when DnaK/J chaperones are absent [29,30]. These results suggest an overlap in substrates between major cytosolic chaperones and PPIs, further reinforcing the importance of PPIs in protein folding. However, now the major task is to identify which of these aggregation-prone proteins require a specific PPI for their in vivo folding. Towards this goal several strategies were undertaken, results of which are further discussed below.

In this work, our major focus has been on the identification of substrate using multiple approaches besides monitoring aggregation of proteins in the absence of PPIs. To identify physiologically relevant client proteins of PPIs, single-amino acid substitutions were introduced in predicted active and substrates-binding domains and used comparative pull-down studies to identify substrates of PpiB, PpiC, FklB and FkpB. Major highlights are: (i) A good correlation exists between aggregation-prone substrates observed in Δ6*ppi* bacteria with interacting partners in pull-down studies. This common set of proteins that aggregate as well as appear as co-purifying proteins with PPIs cover those involved in transcription (RNA polymerase subunits and transcription factors like Rho), translation-associated proteins, particularly enrichment of ribosomal proteins in all sets as well as ribosome-associated GTPases, cell division proteins FtsZ and MinD, the heat shock protein HslU and proteins involved in oxidative stress like subunits of alkyl hydroperoxide reductase (AhpC and AhpF). (ii) Importantly, based on co-purification studies, PpiB emerged with largest number of interacting partners. Interestingly, this interaction was lost with majority of interacting proteins when PpiB PPIase inactive variant PpiB R43A was used as a bait. Our biochemical data clearly show that PpiB R43A mutant quite like its human Cyp18 R55 counterpart lacks the PPIase activity. It has been shown that Cyp18 R55A replacement leads to less than 1% of the PPIase activity [18], which is consistent with our results with PpiB R43A. Thus, we can conclude that interaction of PpiB with majority of its binding partners requires its PPIase activity. (iii) In the case of PpiC, we employed several strategies to identify its substrates such as immunoprecipitations using the single-copy chromosomal 3xFLAG-tagged wild-type PpiC and PpiC F81A, comparative pull-down studies using the His-tagged wild type, active site variant F81A and with a substitution of M57A, and at the proteomic level by comparison between strains lacking all periplasmic PPIs and with additional deletion of the *ppiC* gene. We could show that M57A substitution in the substrate-binding domain [11], while retaining its PPIase activity albeit to a reduced level, can still interact with some potential client proteins but not all. However, PPIase defective F81A in immunoprecipitation experiments does not form the complex with majority of substrates that immunoprecipitated with the wild type. Previously, it had been reported that PpiC interacts with the AhpC protein [11]. In this work, we showed that the PpiC folding catalyst has several substrates including AhpC, AhpF, Ppk, NusG, Ppa, SeqA, Rho, the BipA GTPase, GrxB, FabH, and RbsB. Co-elution of transcription-related proteins (NusG, Rho, SuhB, and RpoA) and those involved in oxidative stress (AhpC/AhpF and OxyR) suggest enrichment of substrates, which are involved in regulation of transcription and oxidative stress as primary clients of PpiC. Quite importantly for PpiC, we could establish a requirement for its interaction with AhpC, but not when the PPIase activity is abrogated. These conclusions draw further support from the measurement of binding affinity using ITC. The dissociation constant with the wild-type PpiC is of the magnitude with *K*_d_ of 47.4 μM with a favorable enthalpy. Furthermore, the negative enthalpy of the interaction (exothermic) of wild-type PpiC with the AhpC peptide, suggests the noncovalent interactions (hydrogen bonds and van der Waals interactions) for its binding. However, of interest is that this interaction between AhpC and PpiC variants M57A (in the substrate-binding site) and PPIase deficient F81A is endothermic, suggesting disruptions of the energetically favorable noncovalent interactions [31]. Thus, PpiC interaction requires its substrate-binding domain as well as the PPIase catalytic domain for its in vivo and in vitro interactions. (iv) Finally, FklB and FkpB show a significant overlap in terms of partner proteins that include ribosomal proteins and the essential sigma factor RpoE.

Pull-down experiments using FklB monomeric variant Y15A revealed reduced substrate interactions as only few proteins were observed to co-elute, although it still retained substantial PPIase activity. These results imply the importance of dimeric shape for binding to its interacting partner proteins. PPIase deficient FklB F198A still showed interacting ability suggesting that the PPIase activity may not be essential for the substrate recognition as has been observed in related FKBP22 from *Shewanella* sp. [20]. However, another FklB variant with W158Y mutation exhibited a reduced PPIase activity and was also impaired in its interaction in pull-down studies. It is likely that W158Y alteration might also cause structural perturbations as has been proposed in the corresponding mutation in *Shewanella* sp. [20]. Thus, some client proteins may need both chaperone-like activity provided via its dimeric structure and while certain other proteins may need both PPIase and chaperone-like activity.

Our work highlights a major role of PPIs in functions related to protein translation process, since many ribosomal proteins either aggregate in the absence of PPIs or co-purify with PPIs. In support of these conclusions, we also show that the EttA protein (Energy-dependent Translational Throttle A) is a substrate of PpiC and FklB, since it was identified during pull-down studies. EttA is a translational factor that has been suggested to modulate the movement of ribosome and tRNA that are required for protein elongation [32]. Similarly, various components of rRNA modification, the RNA helicase DeaD, ribosome assembly factors like BipA and ObgE GTPases were identified as binding partners of various PPIs. BipA and ObgE are known to impact the ribosome assembly [33,34]. Concerning ribosomal proteins such as S2 and S3, they not only aggregate in Δ6*ppi* bacteria, but were also identified as co-eluting partner proteins with PpiB, PpiC, FklB, and FkpB. It had been earlier suggested that ribosomal proteins could specifically be substrates of FkpB [12]. However, our study shows that PPIs exhibit a redundancy so far their interaction with ribosomal proteins is concerned and overall PPIs are intricately related to various components of the translation process.

It is of significance that RpoE and RseA that act as sigma and anti-sigma factors, respectively were identified as specific clients of FkpB and FklB. RpoE and RseA are present in minor quantities in the cell [35] and their amounts and activities are tightly regulated at transcriptional and post-transcriptional level [24,25,36]. RpoE is essential for the cellular viability and regulates transcription of genes whose products are involved in folding and assembly of main cell envelope components like outer membrane proteins and lipopolysaccharide [24,37,38]. We chose to measure the binding affinity of specific peptides derived from RpoE and RseA amino acid sequence with FkpB by ITC, since analysis of peptide coverage in MALDI-TOF analysis of various substrates was much higher for FkpB than with FklB. Additionally, RpoE specifically was observed in the pull-down studies with the wild-type FkpB but not with the active site variant that has lost its PPIase activity. RpoE and the N-terminal domain of RseA are known to physically interact, with specific contacts of N-RseA with the region 2 and the region 4 of RpoE [39]. We had earlier identified essential residues in the conserved region 4 of RpoE and in the N-terminal domain of RseA that are required for their interaction, which is further supported by the co-crystal structure of RpoE and N-RseA [25,27,39]. Thus, specific peptides were chosen that cover such essential domains in RpoE and RseA. Analysis of binding affinities with peptides from the region 2 (−10 binding domain) and the region 4 of RpoE (−35 recognition) and N-RseA domain using ITC studies revealed tight binding with *K*_d_ ranging from 10 μM to 18.9 μM with favorable enthalpy changes suggesting that all complex formations were driven by the establishment of noncovalent interactions (van der Waals contacts, hydrogen bonds). For FkpB, the positive *T*Δ*S* showed that the association was entropically driven, which suggests a burial of solvent-accessible surface area upon binding a scenario similar to what has been observed with cyclophilin-substrate interactions [40]. Consistent with RpoE being a client of PPIs like FkpB and FklB, qRT-PCR analysis showed a reduction in mRNA levels of RpoE regulon members and the *rpoE* gene itself in Δ6*ppi* bacteria as compared to the wild type. This is in contrast to the constitutively up-regulated induction of RpoH regulon members (heat shock regulon) under the same conditions. Since RpoE senses maturation/folding status of OMPs and balance between phospholipids and LPS in the outer membrane (OM), it will be interesting to address, which specific factors are impaired in this signal transduction. If LPS translocation is impacted is under current investigation, since LptB an essential ATPase component of LPS transport system [14] was identified in pull-down studies with PpiC, FkpB and FklB. However, dampening of RpoE induction could as well be due to overall reduction of synthesis/maturation of major abundant OMPs in Δ6*ppi* bacteria.

Although the PPIase activity of all *E*. *coli* PPIs has been measured, yet the relative contribution is unknown. In this work, we thus undertook systematic studies to measure relative contribution of each PPI towards overall cellular enzymatic activity without using recombinant proteins. We could show that PpiB and PpiA are the major contributors of PPIase activity. PpiA and PpiB are homologous proteins located in the periplasm and the cytoplasm, respectively. Although PpiB was found to be the major contributor towards the cytoplasmic PPIase activity, yet a Δ*ppiB* strain exhibits no growth phenotype defect under standard laboratory growth conditions, exhibits wild-type like bacterial motility and cellular morphology and overexpression of the *ppiB* gene also does not confer any phenotypic defect. The lack of any major growth defect is best explained by a significant overlap of substrates with other PPIs as shown in this study with pull-down experiments. Further, we note that a Δ*ppiB* strain JW0514 from deletion set of all ORFs exhibits hypermotility and the cell division phenotype. However, this phenotype was found to be unrelated to PpiB function as transduction of Δ*ppiB* mutation from JW0514 into the wild-type *E*. *coli* confers a wild-type like phenotype for motility and cell morphology and plasmids carrying the wild-type copy of *ppiB* gene do not complement any phenotype of JW0514 (Table S2 and Materials and Methods). Hence, the conclusions concerning the negative regulation of bacterial motility and cell division by PpiB/FklB [41] could not be validated.

In this study, we also constructed a strain lacking all ten PPIs to measure its PPIase activity. However, such a Δ10*ppi* derivative grows extremely poorly. Most importantly, a small residual PPIase activity above the background level was observed. Thus, intensive studies are ongoing to identify potential new PPIs that can explain this residual activity and explain the viability of strains lacking currently known PPIs. It is of interest that several stress-related proteins with an adaptive function, such as polyphosphate kinase (Ppk), the heat shock protein HslU and UspG were identified as putative clients of PPIs [22,42,43], suggesting a co-operation between PPIs and factors in maintaining cellular homeostasis. Furthermore, the identification of several proteins that may require PPIs for their folding or activity has laid a fertile ground for future investigations. Our ongoing studies indeed reveal the PPIase activity-dependent and independent interactions with substrates like FabH, NusG, OxyR, GrxB, YgfZ, Ppk, Ppa, RpoA, ProQ, RapZ, and YrdA (manuscript in preparation).

## 4. Materials and Methods

### 4.1. Bacterial Strains, Plasmids and Media

The bacterial strains and plasmids used in this study are described in supporting Table S2. Luria-Bertani (LB) broth, M9 (Difco) and M9 minimal media were prepared as described [44]. When required, media were supplemented with ampicillin (100 μg/mL), kanamycin (50 μg/mL), tetracycline (10 μg/mL), spectinomycin (50 μg/mL), chloramphenicol (20 μg/mL).

### 4.2. Generation of Null Mutations and the Construction of Δ6ppi Derivatives

Non-polar antibiotic-free deletion mutations of various PPIs-encoding genes or genes whose overexpression restored growth of various Δ6*ppi* strains were constructed by using the λ Red recombinase/FLP-mediated recombination system as described previously [14,45]. The antibiotic cassette was amplified using pKD3 and pKD13 as templates [45]. PCR products from such amplification reactions were electroporated into BW25113 containing the λ Red recombinase-encoding plasmid pKD46 (GK1942). Each deletion was verified by PCR amplification and sequencing of PCR products. Such deletions were transduced into BW25113 wild-type strain by bacteriophage P1-mediated transduction. Multiple null combinations were constructed as described previously, followed by the removal of *aph* or *cat* cassettes using the pCP20 plasmid and confirmed to be non-polar [44,45]. When required, additional deletion derivatives were constructed using *ada* cassette for gene replacement using pCL1920 plasmid as a template in PCR amplification reactions, followed by gene replacement as described above. Transductants were plated in parallel on M9 and LA medium at various temperatures. To validate obtaining of suppressor-free Δ6*ppi* derivatives, deletion derivatives carrying up to five deletion combinations were transformed with either the vector alone or the plasmid carrying the sixth PPI-encoding gene under the control of P_T5_-*lac* promoter. Such six strains then served as a recipient to bring in the last deletion by bacteriophage P1-mediated transductions to construct Δ6*ppi* derivatives and verified as described above. Since deletion derivatives of *ppiB* and *fkpB* genes from the library of gene disruptions in the Keio collection exhibit a polar phenotype, new gene disruptions were constructed using the λ Red-mediated recombineering. In these cases, the *aph* cassettes were introduced using oligonucleotides that contain a ribosome-binding site to allow a constitutive induction of downstream essential genes (*lpxH* in the case of *ppiB* gene and *ispH* in the case of *fkpB* gene, respectively). Furthermore, since the Δ*ppiB* derivative in Keio collection JW0154 exhibits a hypermotility phenotype, this Δ*ppiB* mutation was transduced into wild-type BW25113 and BW30270 strains resulting into SR19818 and SR19840, which exhibited wild-type like cell morphology and wild-type like bacterial motility (Table S2).

### 4.3. Construction of Chromosomal Wild-Type and Single-Copy Mutants of ppiC FLAG Derivatives

To construct chromosomal C-terminal 3xFLAG-tagged genes, pSUB11 plasmid [46] was used as template for PCR amplification as described earlier [14,44]. PCR reactions were carried out using appropriate oligonucleotides. To introduce single amino acid substitutions in the *ppiC* on the chromosome with C-terminal 3xFLAG, mutations were introduced in the forward oligonucleotides. Verification of the introduced mutations in the concerned gene was done on the basis of DNA sequencing of PCR amplification products using chromosomal DNAs as templates.

### 4.4. The Isolation of Aggregated Proteins

Aggregation of cellular proteins was analyzed using isogenic wild-type BW25113 and its Δ6*ppi* derivative. Fifty ml bacterial cultures were grown in M9 medium at 37 °C up to an OD_600_ of 0.3, washed and shifted to LB medium at 30 °C. Cultures were harvested by centrifugation. Pellets were resuspended in 600 µL of B-PER reagent (Pierce, Warsaw, Poland), supplemented with 1 mg/mL lysozyme, a cocktail of protease inhibitors (Sigma, Poznan, Poland), PMSF and 30 U of benzonase and incubated for 90 min on ice with gentle mixing and subjected to fractionation into soluble, membrane and aggregates as described [14]. This procedure is based on previously described method [47] to isolate protein aggregates with modifications that allow better removal of membrane proteins. The pellet fraction containing outer membrane and aggregated proteins were resuspended in 40 µL of 10 mM Tris buffer. Soluble and aggregates were analyzed by gel electrophoresis. To identify protein by MALDI-TOF, 2D electrophoresis was undertaken using 300 μg of protein from aggregate samples. Proteins were incubated in rehydration buffer as per protocol from Bio-Rad (Warsaw, Poland). The mixture was added to hydration tray chamber and incubated in 7 cm immobilized pH gradient (IPG) strip gels. Isoelectric focusing was carried out in PROTEAN i12 (Bio-Rad) IEF cell at 20 °C. For the second dimension, strips were applied to 12.5% SDS-PAGE.

### 4.5. Co-immunoprecipitation with 3xFLAG-tagged PpiC Protein

Isogenic cultures of 3xFLAG-tagged wild-type *ppiC* and its PpiC F81A::3xFLAG derivative (5 mL each) were grown to an OD_600_ of 0.5 and harvested by centrifugation at 12,000× *g* for 20 min at 4 °C. Pellet was resuspended in 100 µL of lysis buffer (Sigma FLAGIPT1), supplemented by a cocktail of protease inhibitors (Sigma), PMSF, benzonase and lysozyme as per manufacturer’s instructions. Samples were incubated with shaking at 4 °C and incubated with 30 µL of washed ANTI-FLAG gel suspension. Samples were shaken for 12 h at 4 °C and centrifuged at 7000× *g* for 30 s and washed. FLAG-fusion protein was eluted under native conditions with 3xFLAG peptide. Equivalent amounts of eluted samples were resolved on a 12% SDS-PAGE and proteins visualized by silver staining.

### 4.6. Protein Purification of Wild-Type and Ppi Mutants

For the protein induction, the minimal coding sequence of *ppi* genes were cloned with either a C-terminal or N-terminal His_6_ affinity tag in the T7 polymerase-based expression vectors (pET24b or pET28b). Specific mutations were introduced by gene synthesis and Gibson cloning. For the purification of hexa-His-tagged PPIs, their cognate genes cloned in pET expression vectors were induced in the *E*. *coli* T7 express derivative lacking all six PPIs (SR21984) at 33 °C at an optical density of 0.1 at 600 nm in a 1 L culture by the addition of either 0.1 mM or 0.3 mM IPTG. Lower concentration of IPTG was used to identify co-eluting proteins, whereas higher concentration of IPTG 0.3 mM to obtain pure proteins. This strain contains a chromosomal copy of the phage T7 RNA polymerase gene, whose expression is controlled by IPTG inducible *lac* promoter and also carries *lacI*^q^ and *lysY* to prevent basal level expression. When only pure protein was needed cultures after 2 h incubation with IPTG, rifampicin was added to prevent the synthesis of host proteins and shaken for another 2 h, as described previously [14]. After the induction, cells were harvested by centrifugation at 12,000 rpm for 30 min. The pellet was resuspended in B-PER reagent (Pierce) and adjusted to contain 50 mM NaH_2_PO_4_, 300 mM NaCl, 10 mM imidazole (buffer A), supplemented with lysozyme to a final concentration of 200 μg/mL, a cocktail of protease inhibitors (Sigma) and 30 units of benzonase (Merck, Poznan, Poland). The mixture was incubated on ice for 45 min with gentle mixing. The lysate was centrifuged at 45,000× *g* for 30 min at 4 °C. Soluble proteins were applied over nickel-nitrilotriacetic acid beads (Qiagen, Geneva Switzerland), washed and eluted with buffer A with a linear gradient (50 mM–500 mM) of imidazole.

### 4.7. PPIase Assay

Total cell lysates were obtained from the wild type, its derivative lacking all four periplasmic PPIs and its isogenic derivatives lacking one of cytoplasmic PPIs or strain lacking all ten PPIs. Cultures (250 mL) were grown at 37 °C in LB or M9 medium up to an OD_600_ of 0.4 and harvested by centrifugation. Pellets were resuspended in B-PER reagent, supplemented with lysozyme to a final concentration of 200 μg/mL, cocktail of protease inhibitors and 30 units of benzonase and incubated on ice for 30 min with mixing. The lysate was centrifuged at 45,000× *g* for 90 min at 4 °C to obtain soluble proteins. For each sample, 10 mg/mL protein was used to measure the PPIase activity in a chymotrypsin-coupled enzymatic assay [48,49] using 8 mM *N*-Suc-Ala-Ala-*cis*-Pro-Phe-*p*-nitroanilide as the test peptide. The PPIase activity was determined in 35 mM HEPES pH 8.0 as assay buffer and the activity was measured at 10 °C. The reaction was initiated by the addition of chymotrypsin (300 μg/mL) and change in absorbance at 390 nm recorded using Specord 200 Plus spectrophotometer equipped with the Peltier temperature control system. For measurement of the PPIase activity with purified individual wild-type or mutant protein, PPIs were used at a concentration of 0.1 μM.

### 4.8. RNA Purification and qRT-PCR Analysis

Isogenic bacterial cultures were grown at either 30 or 37 °C in either M9 minimal medium or LB rich medium in the presence of appropriate antibiotics to an OD_600_ of 0.2. For heat shock, aliquots were immediately shifted to pre-warmed medium held at 43 °C and incubated either for 15 min or 90 min. Samples were collected at times indicated above, and harvested by centrifugation. Pellets were flash frozen in liquid nitrogen and stored at −80 °C. RNA was purified using GenElute Total RNA Purification Kit as per manufacturer instructions for Gram-negative bacteria. RNA was eluted in 50 to 100 μL of DEPC-treated water. RNA amounts were quantified and RNA integrity verified by agarose gel electrophoresis. Moreover, qRT-PCR was used to quantify gene expression changes in Δ6*ppi* mutants as compared to the wild type before and after heat shock treatment. For each target gene, gene-specific primers (Table S3) were used to amplify DNA fragments of ≈100 bp. Purified mRNA (2 μg) was converted to cDNA using iScript Reverse Transcription Supermix from Bio-Rad. qRT-PCR was performed using CFX Connect Real-Time PCR Detection System (Bio-Rad). Reactions were carried out for 40 cycles in an optical 96 well plate with 20 μL reaction volumes containing 10 μL PowerUp SYBR^®^ Green PCR Master Mix (ThermoFisher Scientific, Poznan, Poland), 0.5 μL cDNA, 0.6 μL each of the 10 μM forward and reverse primers, and 8.3 μL of RNase-free water. In addition, samples lacking cDNA in above reaction served as a control for any DNA contamination. Data were analyzed by software Bio-Rad CFX Maestro.

### 4.9. Isothermal Titration Calorimetry (ITC) Measurements

The experiments were conducted using a Nano ITC instrument (TA instruments, Utah USA). Purified proteins were dialyzed against ITC buffer (20 mM sodium phosphate pH 7.5 and 100 mM NaCl). For these experiments, peptides used were used at a concentration of 200  μM in the calorimetric cell and titrated with 2 mM of various protein at 20 °C. Typically for each experiment, 25 injections (2 μL of protein sample) at 200 s intervals were performed. Binding isotherms were analyzed according to 1:1 binding model using NanoAnalyze software.

## Figures and Tables

**Table 1:** The essentiality of peptidyl-prolyl *cis/trans* isomerases (PPIs) in the wild-type strain BW25113 in Luria Agar (LA) medium as revealed by bacteriophage P1-mediated transductional efficiency criteria. Numbers indicate obtained transductants/mL of recipient. Data are from one of the representative experiments with several repeats.

	23 °C		30 °C		37 °C		43 °C	
	M9	LA	M9	LA	M9	LA	M9	LA
Δ(*fklB slyD fkpB tig ppiB*) + vector x Δ*ppiC*	434	8	2369	27	2460	47	−	−
Δ(*fklB slyD fkpB tig ppiB*) + p*ppiC*^+^ x Δ*ppiC*	1740	1630	2280	1973	2710	2432	838	1140
Δ(*fklB slyD tig ppiB ppiC*) + vector x Δ*fkpB*	342	10	1732	37	1562	35	−	−
Δ(*fklB slyD tig ppiB ppiC*) + p*fkpB*^+^ x Δ*fkpB*	2314	2640	1948	2100	1980	2013	954	901
Δ(*fklB slyD tig ppiC fkpB*) + vector x Δ*ppiB*	425	5	1420	24	1830	48	−	−
Δ(*fklB slyD tig ppiC fkpB*) + p*ppiB*^+^ x Δ*ppiB*	1451	1620	1830	1723	2620	2534	1031	977

## Supplementary Materials

The following are available online at https://www.mdpi.com/1422-0067/21/12/4212/s1, Table S1. Proteins that specifically accumulate in aggregation fractions in Δ6*ppi* strains under permissive growth conditions, Table S2. Bacterial strains and plasmids used in this study, Table S3. Primers for qRT-PCR.

## Abbreviations

PPI	peptidyl-prolyl *cis*/*trans* isomerase
FKBP	FK506-binding protein
OMP	outer membrane protein
ITC	isothermal titration calorimetry
