# Supplementary material for: Identification of Substrates of Cytoplasmic Peptidyl-Prolyl Cis/Trans Isomerases and Their Collective Essentiality in Escherichia Coli
Source: Int J Mol Sci. 2020 Jun 13;21(12):4212. doi: 10.3390/ijms21124212 (PMC7353009; doi:10.3390/ijms21124212)
Supplement: Supplementary file 1 [file ijms-21-04212-s001.pdf]

Identification of Substrates of Cytoplasmic Peptidyl-Prolyl *cis/trans* Isomerases and Their Collective Essentiality in *Escherichia coli*

Gracjana Klein *, Paweł Wojtkiewicz †, Daria Biernacka †, Anna Stupak, Patrycja Gorzelak, and Satish Raina *

Unit of Bacterial Genetics, Gdansk University of Technology, Narutowicza 11/12, 80-233 Gdansk, Poland
* Correspondence: gracjana.klein@pg.edu.pl (G.K.); satish.raina@pg.edu.pl (S.R.);
Tel.: +48-58-347-2618 (G.K. & S.R.)
† These authors contributed equally to this work.

Keywords: prolyl isomerase; protein folding; heat shock proteins; protein aggregation; RpoE sigma factor; PpiB; PpiC; FkpB; FklB; AhpC

Supplementary Materials

Table S1. Proteins that specifically accumulate in aggregation fractions in Δ6*ppi* strains under permissive growth conditions

Gene	MW (kDa)	Function	Predicted *cis* conformation of Xaa-Pro	Number of Pro residues
rpoC	155,160	RNA polymerase subunit β′	present	57
rpoB	150,632	RNA polymerase subunit β	present	56
hrpA	149,028	ATP-dependent RNA helicase	present	63
putA	143,815	transcriptional regulator/ proline dehydrogenase	unknown	71
entF	141,991	apo-serine activating enzyme	present	92
purL	141,403	phosphoribosylformylglycinamidine synthetase	present	61
narG	140,489	nitrate reductase A subunit α	present	60
metH	135,997	cobalamin-dependent methionine synthase	present	50
recB	133,959	exodeoxyribonuclease V subunit	unknown	52
dnaE	129,905	DNA polymerase III subunit α	present	54
carB	117,842	carbamoyl phosphate synthetase subunit β	present	44
ileS	104,297	isoleucine-tRNA ligase	present	42
aceE	99,668	pyruvate dehydrogenase	present	36
infB	97,350	translation initiation factor IF-2	unknown	21
gyrA	96,964	DNA gyrase subunit A	unknown	31
acnB	93,498	aconitatase B	unknown	54
lon	87,438	Lon protease	unknown	37
fusA	77,581	elongation factor G	present	33
pnp	77,101	polynucleotide phosphorylase	present	31
glyS	76,813	glycine-tRNA ligase subunit β	present	30
csrD	73,339	regulator of CsrB and CsrC decay	unknown	22
ftsH	70,708	ATP-dependent zinc metalloprotease	present	36

deaD	70,546	ATP-dependent RNA helicase	unknown	30
selB	68,867	selenocysteyl-tRNA-specific translation elongation factor	unknown	27
dxs	67,617	1-deoxy-D-xylulose-5-phosphate synthase	present	35
argS	64,683	arginine-tRNA ligase	present	19
sdhA	64,422	succinate:quinone oxidoreductase	unknown	24
proS	63,693	proline-tRNA ligase	present	28
ptsI	63,562	PTS enzyme I	present	15
ettA	62,443	energy-dependent translational throttle protein	unknown	23
pgi	61,530	glucose-6-phosphate isomerase	unknown	19
oppA	60,899	oligopeptide periplasmic binding protein	present	32
ilvB	60,441	acetohydroxy acid synthase I subunit IlvB	present	37
prfC	59,574	peptide chain release factor RF3	present	20
pgm	58,361	phosphoglucomutase	unknown	28
opgG	57,912	synthesis of membrane-derived oligosaccharides	unknown	32
lysU	57,827	lysine-tRNA ligase (multifunctional)	unknown	19
glpD	56,751	aerobic glycerol 3-phosphate dehydrogenase	unknown	20
glpK	56,231	glycerol kinase	present	14
ahpF	56,177	alkyl hydroperoxide reductase component	present	19
norR	55,236	DNA-binding transcriptional dual regulator	unknown	22
der	55,036	50S ribosomal subunit stability factor	unknown	22
proP	54,846	osmolyte:H^+ symporter	present	23
modF	54,536	ABC family protein	present	22
miaB	53,663	isopentenyl-adenosine A37 tRNA methylthiolase	present	22
otsA	53,611	trehalose-6-phosphate synthase	unknown	26
betB	52,911	betaine aldehyde dehydrogenase	unknown	20
tnaA	52,773	tryptophanase/L-cysteine desulfhydrase	unknown	20
glnG	52,255	DNA-binding transcriptional dual regulator NtrC	present	22
guaB	52,022	inosine 5'-monophosphate dehydrogenase	unknown	16
glnA	51,904	glutamine synthetase	present	27
pykA	51,357	pyruvate kinase II	unknown	14
pykF	50,729	pyruvate kinase I	unknown	12
ffh	49,787	signal recognition particle protein component	present	18
hslU	49,594	ATP-dependent protease ATPase subunit	unknown	17
yegD	49,371	HSP70 family protein	present	22
ygiF	48,389	inorganic triphosphatase	unknown	17
glmM	47,544	phosphoglucosamine mutase	unknown	14
aceA	47,522	isocitrate lyase	unknown	17
purA	47,345	adenylosuccinate synthetase	present	17
rhlB	47,126	ATP-dependent RNA helicase	unknown	22
gatZ	47,109	tagatose-1,6-bisphosphate aldolase 2 subunit	present	18
rho	47,004	transcription termination factor	unknown	17
clpX	46,356	ClpX ATP-dependent protease specific component	present	17
icdA	45,757	isocitrate dehydrogenase	present	19
eno	45,655	enolase	unknown	11
hemL	45,366	glutamate-1-semialdehyde aminotransferase	present	24
tufB	43,314	translation elongation factor Tu 2	unknown	20
tufA	43,284	translation elongation factor Tu 1	unknown	20

entC	42,932	isochorismate synthase EntC	present	27
fabB	42,613	β-ketoacyl-[acyl carrier protein] synthase I	unknown	12
ubiH	42,288	2-octaprenyl-6-methoxyphenol hydroxylase	present	13
tyrA	42,043	fused chorismate mutase	unknown	17
carA	41,431	carbamoyl phosphate synthetase subunit α	unknown	19
prfB	41,251	peptide chain release factor RF2	unknown	7
malK	40,990	maltose ABC transporter ATP binding subunit	unknown	20
ispG	40,684	(*E*)-4-hydroxy-3-methylbut-2-enyl-diphosphate synthase (flavodoxin)	unknown	14
ftsZ	40,324	cell division protein	unknown	15
ybeZ	39,039	PhoH-like protein	unknown	16
ftsX	38,544	cell division protein	present	12
recA	37,973	DNA recombination/repair protein	unknown	10
ruvB	37,174	Holliday junction branch migration complex subunit	present	21
accA	35,242	acetyl-CoA carboxyltransferase subunit α	unknown	16
asnA	36,651	asparagine synthetase A	unknown	14
rpoA	36,512	RNA polymerase subunit α	present	16
lpxC	33,956	UDP-3-*O*-acyl-*N*-acetylglucosamine deacetylase	present	12
amiA	31,412	*N*-acetylmuramoyl-L-alanine amidase A	unknown	9
ppsR	31,211	phosphoenolpyruvate synthetase regulatory protein	present	14
murI	31,002	glutamate racemase	present	19
rsmA	30,420	ribosomal RNA small subunit methyltransferase A	present	20
yafD	29,992	stress responsive	unknown	21
rplB	29,860	50S ribosomal subunit protein L2	present	17
yafJ	28,636	putative glutamine amidotransferase	present	14
gpmA	28,556	2,3-bisphosphoglycerate-dependent phosphoglycerate mutase	unknown	11
zapD	28,292	cell division factor	present	12
modE	28,281	DNA-binding transcriptional dual regulator	unknown	6
panB	28,237	pantothenate biosynthesis	present	1
lpxA	28,080	UDP-*N*-acetylglucosamine acyltransferase	unknown	9
ompR	27,354	transcriptional regulatory protein	present	15
arcA	27,292	DNA-binding transcriptional dual regulator	present	10
nagD	27,163	UMP phosphatase	present	13
artP	27,022	L-arginine ABC transporter ATP binding subunit	unknown	12
purC	26,995	inosine-5′-phosphate biosynthesis	present	7
rpsB	26,744	30S ribosomal subunit protein S2	unknown	7
yigA	26,729	DUF484 domain-containing protein	present	11
trmO	26,362	tRNA m^{6}t^{6}A37 methyltransferase	present	18
rpsC	25,983	30S ribosomal subunit protein S3	present	10
deoD	25,950	purine nucleoside phosphorylase	unknown	6
lolD	24,438	lipoprotein release complex-ATP binding subunit	present	8
can	25,097	carbonic anhydrase 2	unknown	6
rplA	24,730	50S ribosomal subunit protein L1	unknown	7
narL	23,927	DNA-binding transcriptional dual regulator	unknown	9
rcsB	23,671	DNA-binding transcriptional activator	unknown	10
crp	23,640	DNA-binding transcriptional dual regulator	unknown	6
narP	23,575	DNA-binding transcriptional dual regulator	unknown	6

yihA	23,561	GTP-binding protein	unknown	7
rpsD	23,469	30S ribosomal subunit protein S4	unknown	6
pcm	23,258	L-isoaspartate protein carboxylmethyltransferase	present	10
yciO	23,212	putative RNA-binding protein	present	14
evgA	22,690	DNA-binding transcriptional activator	unknown	4
yjaG	22,613	DUF416 domain-containing protein	unknown	5
lexA	22,358	DNA-binding transcriptional repressor	present	9
rplC	22,244	50S ribosomal subunit protein L3	present	6
rplD	22,087	50S ribosomal subunit protein L4	unknown	5
yjgA	21,359	DUF416 domain-containing protein	unknown	7
rfbC	21,270	dTDP-4-dehydrorhamnose 3,5-epimerase	unknown	7
ycfP	21,226	UPF0227 protein	present	8
yaeQ	20,877	uncharacterized protein	unknown	4
wrbA	20,846	NAD(P)H:quinone oxidoreductase	unknown	9
ahpC	20,761	alkyl hydroperoxide reductase	present	8
ycdY	20,724	chaperone protein	unknown	11
seqA	20,315	negative modulator of initiation of replication	unknown	8
rplE	20,302	50S ribosomal subunit protein L5	unknown	6
ybgA	20,211	DUF1722 domain- containing protein	unknown	9
rpsG	20,019	30S ribosomal subunit protein S7	unknown	7
yhbT	19,672	SCP2 domain-containing protein	unknown	7
fabA	18,969	β-hydroxyacyl-acyl carrier protein dehydratase	unknown	7
rplF	18,904	50S ribosomal subunit protein L6	unknown	8
lrp	18,887	DNA-binding transcriptional dual regulator	unknown	7
ubiC	18,777	chorismate lyase	unknown	13
dps	18,695	DNA protection during starvation protein	unknown	2
mug	18,673	stationary phase mismatch/uracil DNA glycosylase	unknown	9
bfr	18,495	bacterioferritin	unknown	1
yajQ	18,344	nucleotide binding protein	unknown	3
smpB	18,269	SsrA-binding protein	present	3
rplJ	17,712	50S ribosomal subunit protein L10	unknown	5
rpsE	17,603	30S ribosomal subunit protein S5	unknown	5
mraZ	17,386	DNA-binding transcriptional repressor	present	6
nrdR	17,229	transcriptional repressor	unknown	5
fabZ	17,033	3-hydroxy-acyl-[acyl-carrier-protein] dehydratase	present	9
ibpB	16,093	small heat shock protein	present	9
rplM	16,019	50S ribosomal subunit protein L13	unknown	6
rpsF	15,703	30S ribosomal subunit protein S6	unknown	5
crl	15,655	RNA polymerase holoenzyme assembly factor	unknown	8
yibN	15,596	putative sulfurtransferase	unknown	5
rplP	15,281	50S ribosomal subunit protein L16	unknown	7
rplO	14,980	50S ribosomal subunit protein L15	present	4
rplK	14,875	50S ribosomal subunit protein L11	present	9
rpsI	14,856	30S ribosomal subunit protein S9	unknown	3
rplQ	14,365	50S ribosomal subunit protein L17	unknown	4
rpsH	14,127	30S ribosomal subunit protein S8	unknown	5
rpsL	13,737	30S ribosomal subunit protein S12	unknown	7
rplN	13,541	50S ribosomal subunit protein L14	unknown	5

rplT	13,497	50S ribosomal subunit protein L20	no Pro	
rplS	13,133	50S ribosomal subunit protein L19	unknown	3
rpsM	13,099	30S ribosomal subunit protein S13	unknown	4
rplV	12,226	50S ribosomal subunit protein L22	unknown	2
rpsN	11,580	30S ribosomal subunit protein S14	unknown	4
rplU	11,564	50S ribosomal subunit protein L21	present	1
rplX	11,316	50S ribosomal subunit protein L24	unknown	3
rpsT	9,684	30S ribosomal subunit protein S20	unknown	1

Table S2. Bacterial strains and plasmids used in this study

Strains/Plasmids	Relevant characteristic	Reference or source
Strains		
BW25113	$lacI^q$ $rrnB_{T14}$ $\Delta lacZ_{WJ16}$ *hsdR514* $\Delta araBAD_{AH33}$ $\Delta rhaBAD_{LD78}$	[45]
T7 Express $lysY/I^q$	MiniF *lysY fhuA2 lacZ::T7 gene1 [lon] ompT*	NEB
GK1942	BW25113 (pKD46)	[14]
SR7760	BW25113 *ppiC<>aph*	This study
SR8331	BW25113 *tig<>aph*	This study
SR8379	BW25113 *slyD<>aph*	This study
JW0026	BW25113 *fkpB<>aph*	[50]
SR9539	BW25113 *fkpB<>aph*	This study
SR15700	BW25113 *fkpB<>ada*	This study
SR20626	BW25113 *ppiB<>ada*	This study
SR20272	BW25113 *tig<>ada*	This study
GK792	BW25113 *tig<>cat*	This study
GK1013	BW25113 *fklB<>aph*	This study
GK1015	BW25113 *ppiB<>aph*	This study
GK4125	BW25113 *fkpB<>aph*	This study
GK4306	Δ*fkpB* $ispH^C$	This study
JW0514	BW25113 *ppiB<>aph*	[50]
SR19818	BW25113 Δ*ppiB* transduced from JW0514	This study
SR19840	BW30270 Δ*ppiB* transduced from JW0514	This study
SR20052	non polar on *lpxH* deletion *ppiB<>aph*	This study
SR18272	BW25113 *ppiB<>frt ppiC<>frt tig<>frt slyD<>frt fklB<>frt*	This study
SR18292	SR18272 *fkpB<>aph* (Δ*6ppi*)	This study
SR21984	T7 Express *ppiB<>frt ppiC<>frt tig<>frt slyD<>frt fklB<>frt fkpB<>frt* (Δ*6ppi*)	This study
SR20072	BW25113 *ppiD<>frt surA<>frt fkpA<>frt ppiA<>frt*	This study
SR20098	SR20072 *ppiC<>aph*	This study
SR20105	SR20072 *fklB<>aph*	This study
SR20155	SR20072 *ppiB<>aph*	This study
SR20457	SR20072 *slyD-<>aph*	This study
SR20273	SR20072 *tig<>ada*	This study
SR20956	SR20273 *fkpB<>aph*	This study

SR20227	BW25113 *ppiA<>aph ppiB<>frt*	This study
GK4591	SR18292 *ppiA<>frt ppiD<>frt fkpA<>frt surA<>cat*	This study
GK4034	SR18292 *fkpB<>frt*	This study
GK3880	BW25113 (*ppiC* wt)::3xFLAG-<>*aph*	This study
GK4394	BW25113 (*ppiC* F81A)::3xFLAG-<>*aph*	This study
Plasmids		
pET24b	expression vector	Novagen
pET28b	expression vector	Novagen
pSR19895	*fkpB*$^+$ in pET24b	This study
pSR19817	*fkpB* N126A in pET24b	This study
pSR19189	*fkpB* P128A in pET24b	This study
pSR19192	*fkpB* N126A H127A in pET24b	This study
pSR19193	*fkpB* E86A in pET24b	This study
pSR18154	*fklB*$^+$ in pET28b	This study
pSR19906	*fklB* Y15A in pET28b	This study
pSR19908	*fklB* F198A in pET28b	This study
pSR19909	*fklB* F198L in pET28b	This study
pSR19911	*fklB* W158Y in pET28b	This study
pSR19873	*ppiB*$^+$ in pET24b	This study
pSR19875	*ppiB* R43A in pET24b	This study
pSR20534	*ppiC*$^+$ in pET24b	This study
pSR20540	*ppiC* F81A in pET24b	This study
pSR20538	*ppiC* H84A in pET24b	This study
pSR20519	*ppiC* M57A in pET24b	This study
pCP20	ts replicon with inducible FLP recombinase	[45]
pKD3	*oriR6K*$_{\gamma}$, *bla*(Amp^R), *kan*, *rgnB*(Ter), *cat*	[45]
pKD13	*oriR6K*$_{\gamma}$, *bla*(Amp^R), *kan*, *rgnB*(Ter)	[45]
pKD46	*araBp-gam-bet-exo*, *bla*(Amp^R), *repA101*(ts)	[45]
pCA24N	IPTG-inducible expression vector cm^R	[51]
JW0027	*ispH*$^+$ in pCA24N cm^R	[51]
JW0514	*ppiB*$^+$ in pCA24N cm^R	[51]
JW4111	*ampC*$^+$ in pCA24N cm^R	[51]
pSUB11	3xFLAG	[46]

References

50. Baba, T.; Ara, T.; Hasegawa, M.; Takai, Y.; Okumura, Y.; Baba, M.; Datsenko, K.A.; Tomita, M.; Wanner, B.L.; Mori, H. Construction of *Escherichia coli* K-12 in-frame, single-gene knockout mutants: the Keio collection. *Mol. Syst. Biol.* **2006,** *2,* 2006.0008. DOI: 10.1038/msb4100050
51. Kitagawa, M.; Ara, T.; Arifuzzaman, M.; Ioka-Nakamichi, T.; Inamoto, E.; Toyonaga, H.; Mori, H. Complete set of ORF clones of *Escherichia coli* ASKA library (a complete set of *E. coli* K-12 ORF archive): unique resources for biological research. *DNA Res.* **2005,** *12,* 291-299. DOI: 10.1093/dnares/dsi012

Table S3. Primers for qRT-PCR

q*dnaK* For	5′-GAC GCA TGG GTC GAA GTT AAA G-3′
q*dnaK* Rev	5′-CGG TTC ACC CAG GTA ATC TTC A-3′
q*rpoE* For	5′-TGG CTG TAT CGG ATT GCT GTA A-3′
q*rpoE* Rev	5′-AGT TTT CAG CTT CAA TGG CAT CC-3′
q*ibpA* For	5′-CGC TTT ACC GTT CTG CTA TTG G-3′
q*ibpA* Rev	5′-AGC AAT GCG GTA ATG GTT TTC G-3′
q*degP* For	5′-GTA GCG ATT GGT AAC CCG TTT G-3′
q*degP* Rev	5′-TCG GTC TGG ATG AAG TTT TCG T-3′
